# Heme oxygenase 1-mediated ferroptosis in Kupffer cells initiates liver injury during heat stroke

**DOI:** 10.1016/j.apsb.2024.05.007

**Published:** 2024-05-13

**Authors:** Ru Li, Riqing Wei, Chenxin Liu, Keying Zhang, Sixiao He, Zhifeng Liu, Junhao Huang, Youyong Tang, Qiyuan An, Ligen Lin, Lishe Gan, Liying Zhao, Xiaoming Zou, Fudi Wang, Yuan Ping, Qiang Ma

**Affiliations:** a The Seventh Affiliated Hospital, Southern Medical University, Foshan 528244, China; b Department of Biopharmaceutics, School of Laboratory Medicine and Biotechnology, Southern Medical University, Guangzhou 510000, China; c Medical Critical Care Medicine, General Hospital of Southern Theatre Command of PLA, Guangzhou 510000, China; d Guangdong Branch Center, National Clinical Research Center for Geriatric Diseases (Chinese PLA General Hospital), Guangzhou 510000, China; e State Key Laboratory of Quality Research in Chinese Medicine, Institute of Chinese Medical Sciences, University of Macau, Avenida da Universidade, Taipa, Macao 999078, China; f School of Pharmaceutical Sciences, Zhejiang Chinese Medical University, Hangzhou 311402, China; g Department of General Surgery, Nanfang Hospital, the First School of Clinical Medicine, Southern Medical University, Guangzhou 510000, China; h The Fourth Affiliated Hospital, the First Affiliated Hospital, School of Public Health, Institute of Translational Medicine, Cancer Center, State Key Laboratory of Experimental Hematology, Zhejiang University School of Medicine, Hangzhou 310000, China; i The First Affiliated Hospital, the Second Affiliated Hospital, Basic Medical Sciences, School of Public Health, Hengyang Medical School, University of South China, Hengyang 421200, China; j College of Pharmaceutical Sciences, Zhejiang University, Hangzhou 310000, China; k Liangzhu Laboratory, Zhejiang University Medical Center, Hangzhou 310000, China; l Guangdong Provincial Key Laboratory of Immune Regulation and Immunotherapy, School of Laboratory Medicine and Biotechnology, Southern Medical University, Guangzhou 510000, China

**Keywords:** Heat stroke, Liver injury, Kupffer cells, Ferroptosis, Heme oxygenase 1, Phosphatidylinositol 4-kinase beta, NLRP3, Early growth response factor 1

## Abstract

With the escalating prevalence of global heat waves, heat stroke has become a prominent health concern, leading to substantial liver damage. Unlike other forms of liver injury, heat stroke-induced damage is characterized by heat cytotoxicity and heightened inflammation, directly contributing to elevated mortality rates. While clinical assessments have identified elevated bilirubin levels as indicative of Kupffer cell dysfunction, their specific correlation with heat stroke liver injury remains unclear. Our hypothesis proposes the involvement of Kupffer cell ferroptosis during heat stroke, initiating IL-1*β*-mediated inflammation. Using single-cell RNA sequencing of murine macrophages, a distinct and highly susceptible Kupffer cell subtype, Clec4F^+^/CD206^+^, emerged, with heme oxygenase 1 (HMOX-1) playing a pivotal role. Mechanistically, heat-induced HMOX-1, regulated by early growth response factor 1, mediated ferroptosis in Kupffer cells, specifically in the Clec4F^+^/CD206^+^ subtype (KC2), activating phosphatidylinositol 4-kinase beta and promoting PI4P production. This cascade triggered NLRP3 inflammasome activation and maturation of IL-1*β*. These findings underscore the critical role of targeted therapy against HMOX-1 in ferroptosis within Kupffer cells, particularly in Clec4F^+^/CD206^+^ KCs. Such an approach has the potential to mitigate inflammation and alleviate acute liver injury in the context of heat stroke, offering a promising avenue for future therapeutic interventions.

## Graphical abstract

This study elucidates the intricate molecular mechanisms underpinning heat stroke-induced liver injury, highlighting the diverse responses of Kupffer cell (KC) subsets and the pivotal role of HMOX-1 in orchestrating ferroptosis in KCs, particularly in KC2, and NLRP3 inflammasome activation.

**Figure:**
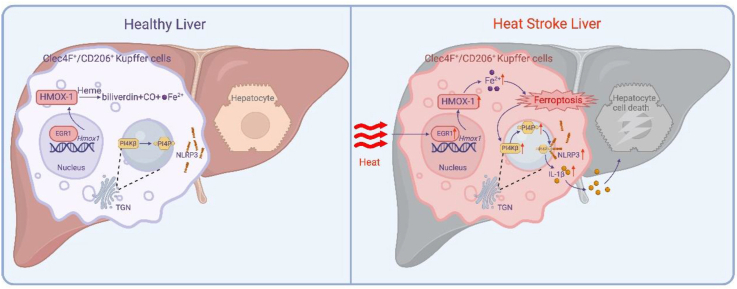

## 1 Introduction

Heat stroke (HS) is a critical medical condition characterized by severe hyperthermia (>40.5 °C) accompanied by central nervous system dysfunction and multiple organ failure^1^. Liver injury is a frequently documented complication of HS and a direct contributor to mortality in affected patients^2^^,^^3^. Given the liver's crucial role in metabolism, immunity, and regulation, it acts as a perpetrator rather than a victim in the host response failure and multi-organ damage. Unlike other liver injuries associated with bacterial invasion, viral replication, or drug metabolism, HS-induced liver injury, as a sterile inflammatory injury, is uniquely caused by cellular thermotoxic effects and intense inflammatory responses^4^^,^^5^.

In our HS patients, elevated serum levels of alanine aminotransferase (ALT), aspartate aminotransferase (AST), and total bilirubin (TBIL) indicate severe liver damage. Kupffer cells (KCs), responsible for hemoglobin degradation and subsequent bilirubin generation and transport^6^, exhibit impaired function during HS, implicating their involvement in liver injury. Positioned within hepatic sinusoids, KCs modulate immune tolerance and produce inflammatory factors, such as IL-1*β*, making them primary responders to inflammation by up-regulating chemokines that recruit immune cells to the lesion site^7^, ^8^, ^9^. Yet, the exact role and mechanisms of KCs in initiating IL-1*β* in response to HS are not well understood. NOD-like receptor family pyrin domain containing 3 (NLRP3) inflammasomes typically regulate IL-1*β* maturation and release, triggering pyroptosis^10^, ^11^, ^12^. KCs, as resident immune cells, may undergo alternative forms of cell death, including apoptosis, necrosis, or ferroptosis^13^, ^14^, ^15^.

Ferroptosis, a lipid peroxidation-driven, iron-dependent, regulated form of cell death, is closely associated with iron overload and the generation of lipid peroxides^16^. Despite playing a crucial role in ischemia-reperfusion, viral hepatitis, liver fibrosis, nonalcoholic steatohepatitis, and fatty liver disease-induced liver injury, there is currently no research reporting the detrimental effects of ferroptosis in HS-induced liver injury^17^, ^18^, ^19^, ^20^, ^21^, ^22^. Heme oxygenase 1 (HMOX-1), a protein crucial in iron overload during ferroptosis and expressed in KCs, plays a significant role in heme breakdown^23^^,^^24^. Although HMOX-1 is generally up-regulated in liver damage processes, its excessive expression in KCs during HS promotes liver damage, and the underlying mechanism remains unclear^25^, ^26^, ^27^.

This study aims to elucidate ferroptosis mechanisms in KCs and their contribution to initiating inflammatory responses leading to liver injury in HS mice. Observations indicate that KCs initiate IL-1*β* production early in HS recovery, preceding monocyte-derived macrophages. KCs predominantly undergo ferroptosis rather than pyroptosis or other forms of cell death under heat treatment. Single-cell RNA sequencing and specific *Hmox1* knockout identify HMOX-1 as a mediator of ferroptosis, particularly in the KC subtype KC2 in HS mice, leading to NLRP3 inflammasome activation. These findings provide insights into ferroptosis-triggered NLRP3 inflammasome activation, laying the foundation for targeted therapy against HMOX-1 in the KCs subtype of HS-induced liver injury.

## 2 Materials and methods

### 2.1 Details of individuals with HS and healthy controls

This single-center retrospective cohort study included patients diagnosed with severe heat stroke admitted to the ICU of the General Hospital of the Southern Theater Command of PLA between October 2008 and May 2019. This study was approved by the Ethics Committee of the General Hospital of the Southern Theater Command (Number: Hospital Ethics [2020]-9), and the need to obtain informed consent was waived. More detailed information is provided in Supporting Information Tables S1 and S2.

### 2.2 Mice

Male C57BL/6 wild-type (WT) mice, aged 8–9 weeks and specifically pathogen-free, were obtained from the Animal Center of Southern Medical University (Guangzhou, China). The mice were housed in groups of four per cage under controlled environmental conditions: 12-h light–dark cycle, 55 ± 5% relative humidity (RH), and an ambient temperature of 25 °C. They had ad libitum access to standard chow and water throughout the study period. All experimental procedures involving animals were conducted following the ethical guidelines provided by the National Institutes of Health for the care and use of experimental animals. The study protocol was approved by the Animal Care and Use Committee of Nanfang Hospital (License No. NFYY-2020-0939). To induce HS, mice were placed in an artificial climate chamber set to a temperature of 39.5 °C and a relative humidity of 65%. The onset of HS was determined when the mice's core body temperature (*T*_c_) reached approximately 42.7–43 °C or when signs of exhaustion were observed. Following the HS experiment, mice were returned to their normal feeding environment. According to the core body temperature curve of HS mice, we chose to collect samples at 0, 6, and 24 h after the onset of HS. At 0 h, mice were in the initial stage of heat stroke. At 6 h, their body temperature began to decrease rapidly and remained low, while at 24 h, the body temperature slightly rose but remained in a mild fever state. Therefore, these three-time points can reflect different physiological states of heat stroke and are crucial for our study.

Specific Treatments:

Clodronate Liposomes (CLP) Treatment: CLP (200 μL) was injected into the tail vein of 20–25 g mice 7 days before HS. The control group received an equivalent dose of Control Liposomes (CTR). HS was conducted on the seventh day after administration.

Vitamin E (VE) Administration: Mice were orally given VE at a dosage of 500 mg/kg for 7 days before HS.

Liproxstatin-1 Treatment: Liproxstatin-1 was administered *via* intraperitoneal injection at a dose of 10 mg/kg, given 2 h before HS.

Deferoxamine (DFO) Administration: DFO was administered through intraperitoneal injection at a dosage of 100 mg/kg for 7 days before HS.

Zinc Protoporphyrin (ZnPP) Treatment: ZnPP was given *via* intraperitoneal injection at a dose of 10 mg/kg, 24 h before HS.

Cobalt Protoporphyrin (CoPP) Administration: CoPP was administered through intraperitoneal injection at a dosage of 5 mg/kg, given 24 h before HS.

Genetic Modifications:

Mice with specific knockout of *Hmox1* in KCs were generated by crossing C57BL/6J-*Clec4f*^*em1(cre)Glass*^/J (033296, Jackson lab) with C57BL/6J-*Hmox1*^*em1Cflox*^/Cya (S–CKO-02911, Cyagen Biosciences) mice, resulting in *Clec4f-cre*x*Hmox1*^*flox/flox*^ mice.

Mice with a knockout of *Nlrp3* were purchased from Jackson Laboratory (021302, B6.129S6-*Nlrp3*^*tm1Bhk*^/J).

### 2.3 Cell culture

The cells were usually cultured in Dulbecco's modified Eagle's medium (DMEM) supplemented with 10% fetal bovine serum (164210, Procell) and penicillin–streptomycin mixture (P1400, Solarbio). Cells were initially obtained from the American Type Culture Collection (ATCC) and regularly monitored for contamination of other cell types. All cell cultures were maintained at 37 °C in an environment containing 5% CO_2_ (*v*/*v*).

In preparation for *in vitro* heat stress, the cells were pre-cultured under the following conditions. To mimic the *in vivo* heat treatment conditions, we subjected the cells to a heat shock at 43 °C for 3 h in a cell culture incubator and then allowed them to recover at 37 °C for 0, 6, or 24 h.

Cells were initially cultured with reduced serum levels (2% serum).

Cells were treated with Nec1 (30 μmol/L) for 1 h.

Cells were treated with 3-MA (100 μmol/L) for 1 h.

Cells were treated with z-vad-fmk (10 μmol/L) for 1 h.

Cells were treated with Disulfiram (30 μmol/L) for 1 h.

Cells were treated with Fer-1 (1 μmol/L) for 1 h.

Cells were treated with ZnPP (4 μmol/L) for 12 h.

Cells were treated with CoPP (4 μmol/L) for 12 h.

Cells were treated with PI-273 (5 μmol/L) for 1 h.

Cells were treated with a PI4K*β* inhibitor (5 μmol/L) for 1 h.

### 2.4 Liver hepatocyte and macrophage extraction

Mice were intraperitoneally anesthetized with 1% pentobarbital sodium at a dose of 50 mg/kg. The liver was then carefully extracted, and liver-releasing cells were gently disaggregated using ophthalmic forceps in a 0.064% collagenase IV solution preheated to 37 °C. The resulting cell suspension was filtered over ice through a 100 μm diameter filter into 30 mL of HBSS-CaCl_2_ buffer. Centrifugation at 50 × *g* for 3 min at 4 °C yielded a hepatocyte-enriched precipitate. Hepatocytes were further processed for downstream experiments.

Hepatocytes were resuspended in 30 mL of cold HBSS-CaCl_2_ buffer, followed by an additional round of centrifugation at 50 × *g* for 3 min at 4 °C. After discarding the supernatant, hepatocytes were resuspended in 20 mL of cold HBSS-CaCl_2_ buffer, underwent further washing, and then resuspended in 10 mL of DMEM complete medium containing 10% FBS for subsequent cell culture. Post-culture, cells were washed with PBS before being utilized in subsequent experimental procedures.

To obtain macrophages, 25% and 50% Percoll solutions were prepared. A 50 mL centrifuge tube was loaded with 10 mL of 50% Percoll and 20 mL of 25% Percoll. Filtered supernatant (10 mL) with a 100 μm diameter was collected and added to the 25% and 50% Percoll solutions. Centrifugation at 1200 × *g* for 30 min at 4 °C separated cells, which were then mixed with an equal volume of PBS and centrifuged at 400 × *g* for 10 min at 4 °C. The cells were washed and resuspended in PBS. Macrophages were identified by flow cytometry using specific antibodies including 10 μL of PerCP Cyanine 5.5 anti-CD45, 10 μL of PE anti-CD19, 10 μL of APC/Cyanine 7 anti-CD3, 10 μL of PE/Cyanine 7 anti-Ly6G, 10 μL of FITC anti-CD11b, and 10 μL of APC anti-F4/80. CD45^+^/CD19^–^/CD3^–^/Ly6G^–^/CD11b^+^/F4/80^+^ macrophages were sorted. Cell viability, determined using trypan blue staining, ensured viability exceeding 90%.

The isolated cells were washed with cold PBS containing 0.1% bovine serum albumin (BSA). Single-cell RNA sequencing analysis was performed using the Chromium System (10 × Genomics, CA, USA).

For KC2 cell isolation, PerCP Cyanine 5.5 anti-CD45, AF647 anti-Clec4F, and FITC anti-CD206/Alexa Fluor® 700 anti-CD206 antibodies were used for screening to obtain CD45^+^/Clec4F^+^/CD206^+^ KC2.

### 2.5 Co-culture

Primary hepatocytes or AML12 liver cell line (1 × 10^5^/mL) were seeded into 24-well culture plates, and subsequently, 0.25 × 10^5^ KCs or ImKC were cultivated on transwell inserts with a pore size of 0.4 μm for 24 h. The experimental group was exposed to a 43 °C temperature for 3 h and then allowed to recover at 37 °C for 6 h. After this, the supernatant or cells were collected for further analysis.

### 2.6 Single-cell RNA sequencing

Single-cell suspensions (2 × 10^5^ per mL) in PBS (HyClone) were loaded onto a microwell chip using the Singleron Matrix Single Cell Processing System. Barcoding beads were subsequently collected from the microwell chip. Reverse transcription of the mRNA captured by the barcoding beads was performed to obtain cDNA, followed by PCR amplification. The amplified cDNA was then fragmented and ligated with sequencing adapters. scRNA-seq libraries were constructed according to the GEXSCOPE Single Cell RNA Library Kits (Singleron) protocol. Individual libraries were diluted to 4 nmol/L, pooled, and sequenced on Illumina novaseq 6000 with 150 bp paired-end reads.

Raw reads were processed to generate gene expression profiles using CeleScope v1.1.4 (Singleron Biotechnologies) with default parameters.

Seurat v 3.1.2 was used for quality control, dimensionality reduction, and clustering. For each sample dataset, the expression matrix was filtered by the following criteria: 1) cells with a gene count less than 200 or with a top 5% gene count were excluded; 2) cells with a top 5% UMI count were excluded; 3) cells with mitochondrial content >20% were excluded; 4) genes expressed in fewer than 5 cells were excluded. The gene expression matrix was normalized and scaled using the functions NormalizeData and ScaleData. The top 2000 variable genes were selected by FindVariableFeatures for PCA analysis. Cell clusters were visualized using t-Distributed Stochastic Neighbor Embedding (t-SNE) with Seurat functions RunTSNE.

To identify differentially expressed genes (DEGs), the Seurat FindMarkers function based on the Wilcoxon rank sum test with default parameters was used. Genes expressed in more than 10% of the cells in both of the compared groups of cells and with an average log (Fold Change) value greater than 0.25 were selected as DEGs. The adjusted *P*-value was calculated by Bonferroni correction, and a value of 0.05 was used as the criterion to evaluate statistical significance.

### 2.7 ALT/AST/TBIL assay

Plasma ALT/AST/TBIL activity was determined using the commercially available AST/ALT Assay Kit (Nanjing Jiancheng Bioengineering Institute) and TBIL Assay Kit (Changchun Huili Biotech) according to the manufacturer's instructions.

### 2.8 Flow cytometric analysis

Disaggregated single cells isolated from the liver of HS mice were labeled with APC anti-F4/80 (ab105080, Abcam), FITC anti-CD11b (ab24874, Abcam), PerCP/Cyanine5.5 anti-CD45 (103132, Biolegend), AF647 anti-Clec4F (156804, Biolegend), FITC anti-CD206 (141704, Biolegend), PE anti-HMOX-1 (ab83214, Abcam). Cell death was analyzed by PI (ST511, Beyotime) staining.

### 2.9 BODIPY 581/591 C11 imaging assay

In the study, frozen sections of the livers from HS mice at various recovery time points were prepared for imaging. Before imaging, frozen sections were co-treated with 5 μmol/L of BODIPY 581/591 C11 (D3861, ThermoFisher) dye for 30 min at 37 °C incubator. Images were acquired on a fluorescence microscope (Life) at 563 nm for the reduced form BODIPY-C11, and 488 nm for the oxidized form.

### 2.10 4-Hydroxynonenal (4-HNE) immunohistochemical staining

Paraffin-embedded tissues were sectioned at 4 μm thickness, routine immunohistochemical staining was performed using antibody 4-HNE, and photo collection was performed under a light microscope.

### 2.11 DAB enhanced Perls’ Prussian Blue staining

Paraffin-embedded tissue sections (4 μm thickness) underwent DAB-enhanced Perls’ Prussian Blue staining. After dewaxing and water washing, sections were immersed in a Perls staining solution (2.5% potassium ferrocyanide, 2.5% HCl, 1:1 ratio) for 30 min, followed by distilled water rinse. To block endogenous peroxidase, sections were treated with 3% H_2_O_2_ methanol solution for 30 min, then rinsed with 0.01 mol/L PBS three times for 5 min each. Coloration was achieved with a 10-min application of DAB color solution (0.025% DAB, 0.003% H_2_O_2_), and nuclei were lightly stained with nuclear fast red for 10 min. After tap water rinse, sections were dehydrated, transparentized, and sealed with neutral gum.

### 2.12 Transmission electron microscopy

Mitochondrial changes were measured under a transmission electron microscope, as previously described^28^.

### 2.13 Tyramide signal amplification (TSA)-based immunofluorescent multiplex

Multicolor immunofluorescence analyses were conducted on 4-μm-thick sections of formalin-fixed paraffin-embedded (FFPE) tissues. The slides underwent deparaffinization in xylene and were subsequently hydrated through a series of decreasing graded ethanol solutions. Heat-induced antigen retrieval was carried out using EDTA Antigen Retrieval Solution (pH = 9.0). Sections were initially treated with a 3% H_2_O_2_ methanol solution for 30 min to eliminate endogenous peroxidase in tissues and cells. Subsequently, they were blocked using 5% goat serum in phosphate-buffered saline (PBS) and then co-stained with antibodies recognizing Clec4F (AF2784, R&D), CD206 (ab64693, Abcam), HMOX-1 (ab52947, Abcam), NLRP3 (MAB7578, R&D), IL-1*β* (AF-4-1-NA, R&D), and DAPI.

For immunofluorescence detection on cell slides, they were washed with PBS three times, fixed with 4% paraformaldehyde for 20 min, and then treated with 3% H_2_O_2_ at room temperature for 10 min to remove endogenous peroxidase. The cells were blocked with 0.25% Triton X-100 in PBS for 10 min at room temperature and subsequently with 5% goat serum in PBS. They were then co-stained with antibodies recognizing NLRP3 (ab4207, Abcam), TGN46 (ab16059, Abcam), PI4K*β* (54210, SAB), EGR1 (4153S, Cell Signaling Technology), and HMOX-1 (ab52947, Abcam) at 4 °C overnight. The following day, the slides were washed with PBS and incubated with peroxidase-labeled goat anti-rabbit IgG polymer (PV-6001, ZSGB-Bio) for 30 min at room temperature (22 °C). A TSA indirect kit (Jilin Histova) was employed following the manufacturer's instructions. After washing, the nuclei were counterstained with DAPI. Images were captured using a confocal microscope (Olympus), and image analysis was performed using ZEN Microscopy Software.

### 2.14 Measurement of malondialdehyde (MDA) in liver

MDA in different liver tissues was determined using the commercially available MDA Assay Kit (S0131M, Beyotime) according to the manufacturer's instructions.

### 2.15 Measurement of tissue non-heme iron

Tissue non-heme iron changes were measured using the chromogen method as previously described^29^.

### 2.16 MitoSOX imaging assay

Miro ROS of cells was assessed using the commercially available MitoSOX Red (M36008, Thermo) following the manufacturer's guidelines. In brief, the treated cells were exposed to 500 nmol/L MitoSOX with HBSS with calcium and magnesium and allowed to incubate in a 37 °C incubator for 30 min, and then subjected to analysis using a confocal microscope.

### 2.17 FerroOrange imaging assay

Fe^2+^ of cells was assessed using the commercially available FerroOrange (F374, Dojindo) following the manufacturer's guidelines. In brief, the treated cells were exposed to 1 μmol/L FerroOrange with HBSS and allowed to incubate in a 37 °C incubator for 30 min, and then subjected to analysis using a confocal microscope.

### 2.18 Measurement of Liperfluo

Lipid peroxidation in various cell samples was assessed using the commercially available Liperfluo Dye (L248, Dojindo) following the manufacturer's guidelines. In brief, the treated cells were exposed to 1 μmol/L Liperfluo and allowed to incubate in a 37 °C incubator for 30 min. Following this, the cells were washed with PBS three times and then subjected to analysis using flow cytometry.

### 2.19 Measurement of total iron in cells

The total iron of different cells was determined using the commercially available Intracellular Iron Colorimetric Assay Kit (E1042, Applygen) according to the manufacturer's instructions.

### 2.20 Quantitative real-time polymerase chain reaction (qRT-PCR)

Total RNA was extracted from the cells employing an RNA Extraction Reagent (G3326-15, Servicebio), following the provided protocol. Reverse transcription of mRNA to cDNA was performed using SweScript All-in-One RT SuperMix (G3337-100, Servicebio). The mRNA expression levels were assessed using 2 × Universal Blue SYBR Green qPCR Master Mix (G3326-15, Servicebio), and subsequently, the data were normalized to the GAPDH mRNA level. Specific primer sequences are available in the Supporting Tables.

### 2.21 Gene identification in gene knockout mice

For gene identification in knockout mice, tail DNA was isolated using the commercially available Mouse Direct PCR Kit (B40015, Bimake) as per the manufacturer's guidelines. Subsequently, PCR and agarose gel electrophoresis were carried out based on the specific gene identification procedures for each gene knockout mouse.

### 2.22 Measurement of mouse interleukin 1β (IL-1β)

Mouse plasma, collected with EDTA, was centrifuged (15 min, 1000 × *g*, 4 °C). IL-1*β* was measured using the Mouse IL-1*β* ELISA Kit (CSB-E08054m, CUSABIO), following the manufacturer's protocol.

### 2.23 Western blotting analysis

Tissues and cells were lysed in total protein lysis buffer (P0013, Beyotime), and protein concentration in the supernatant was determined with a BCA protein detection kit (DQ111-01, TransGen). Western blotting utilized primary antibodies against the following targets: HMOX-1 (ab52947, Abcam), EGR1 (4153S, Cell Signaling Technology), PI4K*β* (54210, SAB), and caspase-1 (ab179515, Abcam). After incubation with a peroxidase-conjugated secondary antibody (1:10,000), the immunoreactive protein band signal was visualized using enhanced chemiluminescence, following the manufacturer's guidelines.

### 2.24 Plasmids, viruses, and stable cell lines

The vectors for knockdown or overexpression were custom-synthesized, and cells were transfected with specific constructs. The PI4P probe with EGFP or mCherry tags (OSBP-PH-GFP/mCherry, NM_001033174, 89–187 aa) was synthesized by VectorBuilder (VB220523-1449 yne) and TsingkeBiotechnology Co., Ltd. (GZ0054531), respectively. TsingkeBiotechnology also provided the following constructs: *Hmox1* CDS region, *sh-Hmox1* (ACAGTGGCAGTGGGAATTTAT, sh0411-2696), *sh-PI4K2A* (CCCAAGAATGAAGAGCCATAT, sh0406-2505), *sh-PI4Kβ* (CCTCAAAGAGAGGTTCCACAT, sh1015-8767), and *sh-Egr1* (CATCGCTCTGAATAATGAGAA, sh0411-2692). A blank vector from the same source served as a control. The generated vector was transfected into HEK-293T cells along with LIPO 8000 (C0533, Beyotime) and helper plasmids pSPAX2 and pMD2.G to produce a viral solution. The viral solution was centrifuged, filtered, and used to infect target cells. Sorting was performed by Flow Cytometry 48–96 h later, or cells were treated with 6 μg/mL puromycin (1299MG025, Biofroxx) or 1 mg/mL neomycin (N6063-25g, Macklin) for 48 h to establish stable expression.

### 2.25 Chromatin immunoprecipitation (ChIP) assay

To explore EGR1's binding region on the *Hmox1* promoter, we conducted ChIP Experiments (abs50034, Absin) following the provided guidelines. In brief, formaldehyde (37%) was added to cells for cross-linking and then terminated using glycine. Cell scraping and sonication were performed to generate fragmented chromatin. The supernatant was used for immunoprecipitation. Three reaction tubes were set up: a positive control antibody tube, a negative control IgG tube, and the EGR1 antibody tube. After incubation, protein A/G magnetic beads were added, and the protein–DNA complex was eluted and decrosslinked. DNA purification was done, and quantitative real-time PCR was employed to analyze the enriched region within the *Hmox1* promoter.

### 2.26 Dual-luciferase reporter assay

The wild-type (WT) and mutated *Hmox1* promoter sequences were synthesized and inserted into the pEZX-FR03-Basic vector from GeneCopoeia. ImKC cells were transfected with either an empty vector (control), the WT *Hmox1* promoter plasmid, or the mutated *Hmox1* promoter plasmid (1.5 μg per well). Following transfection, cells were lysed and assessed using the Luc-Pair™ Duo-Luciferase HS Assay Kit from Genecopoeia (LF004). Luciferase activity was quantified using a Varioskan LUX multimode microplate reader from Thermo Fisher Scientific.

### 2.27 Statistical analysis

The data are presented as mean values ± standard error of mean (SEM) and individual data points (representing individual mice) are included in all graphs to demonstrate the distribution. Statistical significance was determined using GraphPad Prism9 software and one-way ANOVA with Tukey's *post hoc* test. The survival rate significance was assessed using the log-rank (Mantel–Cox) test. The figure legends provide details on the number of samples or mice per group, the replicates in independent experiments, and the specific statistical tests used. For *in vivo* studies, mice were randomly assigned to treatment groups. A *P*-value of <0.05 was considered to be statistically significant.

## 3 Results

### 3.1 Kupffer cell ferroptosis drives liver injury in heat stroke mice

**Figure 1:**
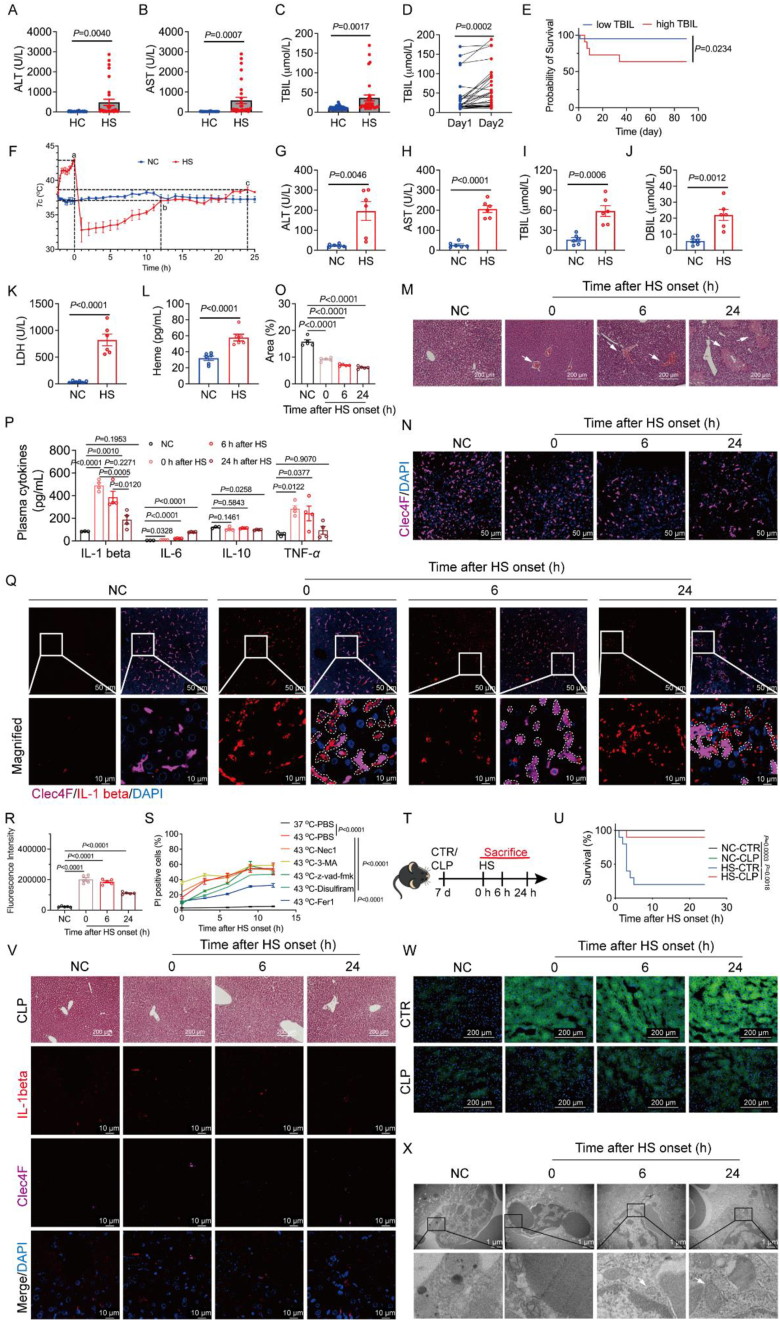
Key role of KCs ferroptosis in HS mice mortality. (A) ALT, (B) AST, and (C) TBIL levels in serum from HC (*n* = 32) and HS (*n* = 32) individuals. (D) TBIL levels in the serum of human HS patients on the first- and second-days. (E) Survival time of HS patients was categorized by low (0–23 μmol/L) and high (>23 μmol/L) serum TBIL levels on the first day. (F) Core body temperature (*T*_c_) of HS mice during HS and the recovery phase (*n* = 4 mice per group). (G) ALT, (H) AST, (I) TBIL, (J) DBIL, (K) LDH, and (L) Heme levels in the plasma of HS mice recovery 24 h (*n* = 6 mice per group). (M) Representative histological images of liver paraffin sections stained with Hematoxylin and Eosin (H&E) (scale bar: 200 μm). (N) Representative immunofluorescence staining images of KCs labeled with Clec4F (purple) and DAPI (blue) in the liver of mice after HS (scale bar: 50 μm), with statistical results of quantification of Clec4F fluorescent positive regions (*n* = 4) (O). (P) Plasma IL-1*β*, IL-6, IL-10, and TNF-*α* content measurement by ELISA (*n* = 3–4 mice per group). (Q) Representative immunofluorescence staining images labeled with IL-1*β* (red), Clec4F (purple), and DAPI (blue) in the liver of mice after HS, depicting co-localization (scale bar: 50 μm), with statistical results of quantification of IL-1*β* fluorescent positive regions (*n* = 5) (R). (S) Cell death detection (PI positive) in KCs pretreated with different inhibitors followed by heat stress (*n* = 3). (T) Schematic diagram illustrating the determination of KCs depletion through Clodronate Liposomes (CLP) and Control Liposomes (CTR). (U) Survival curves of mice pretreated with 200 μL CTR or CLP followed by HS on Day 7 (*n* = 15 mice per group). (V) Representative histological images of liver paraffin sections from HS mice after KCs depletion stained with H&E (scale bar: 200 μm) and labeled with IL-1*β* (red), Clec4F (purple), and DAPI (blue) (scale bar: 10 μm) in the liver. (W) Representative images of BODIPY 581/591 C11 staining in liver tissue of HS mice pretreated with CLP or CTR (scale bar: 200 μm). (X) Representative images from the transmission electron microscope showing mitochondrial changes in the liver of HS mice, with white arrows indicating smaller mitochondria, crest breaks, and deep staining (scale bar: 1 μm). Data are presented as the mean ± SEM. Paired *t*-tests were used in TBIL of Day 1 and Day 2 experiments. Significance was assessed by the Student *t* test or one-way ANOVA with Tukey's *post hoc* test. Significance in (E) and (U) determined log-rank (Mantel–Cox) test.

To evaluate the severity of liver damage in HS patients, we examined serum levels of ALT, AST, and TBIL, revealing a significant elevation in 32 HS individuals compared to 32 healthy controls (HC) (Fig. 1A–C). Continuous monitoring of bilirubin levels on the first- and second-days post-HS onset demonstrated a sustained increase (Fig. 1D). We categorized HS patients into low (0–23 μmol/L) and high (>23 μmol/L) TBIL groups to explore the correlation with survival time. The analysis revealed a significantly lower survival rate in the high bilirubin group (Fig. 1E).

In an HS mouse model, we observed an average time of 2.25 h for mice to reach 43 °C, followed by a fluctuating body temperature during the recovery phase (Fig. 1F). Consistent with our finding in patients with HS, we observed that at 24 h of recovery, elevated plasma levels of liver injury markers, such as ALT, AST, TBIL, direct bilirubin (DBIL), lactate dehydrogenase (LDH), and heme were observed in HS mice compared to controls (Fig. 1G–L). Examination of mouse liver tissue at different recovery time points revealed hepatocyte swelling and necrosis, worsening during the recovery period (Fig. 1M).

Analysis of blood specimens revealed elevated levels of TBIL, correlating with shortened patient survival time. This suggests a potential role for KCs in HS, given their involvement in bilirubin production and transport^6^. KC numbers significantly decreased in HS mice compared to controls (Fig. 1N–O). IL-1*β*, an inflammatory factor, was elevated in plasma at 0 h, and its expression in the liver tissue co-localized with the KCs marker Clec4F at HS onset (Fig. 1P–R). M1 macrophages and monocytes appeared later in the recovery phase, suggesting their differentiation from KCs (Supporting Informatin Fig. S1A–S1D). In a co-culture system, KCs exhibited increased heat-induced cell death, contributing to hepatocyte death, implicating KC death in HS-related liver injury (Fig. S1E and S1F). Ferroptosis was identified as a key contributor to heat-induced KCs cytotoxicity in co-culture systems, as demonstrated by reduced hepatocyte death with Ferrostatin-1 (Fer-1) treatment (Fig. 1S, Fig. S1G).

Depleting liver macrophages with Clodronate Liposomes (CLP) reduced liver injury, IL-1*β*, and improved survival in HS mice (Fig. 1T–V, Fig. S1H–S1K), supporting the role of KCs death in HS-related liver injury. Further investigations revealed increased liver injury with oxidative stress, as shown by lipid peroxidation (Fig. 1W). Transmission electron microscopy revealed KCs mitochondria damage (Fig. 1X), while MDA and tissue non-heme iron levels decreased after KCs depletion (Fig. S1L–S1O). Pretreatment with ferroptosis inhibitors vitamin E (VE), liproxstatin-1, and deferoxamine (DFO) increased survival rates and reduced liver injury (Supporting Information Fig. S2A–S2C). Prussian blue, staining for free iron, Malondialdehyde (MDA), tissue non-heme iron, and lipid peroxidation also showed decreased levels (Fig. S2D–S2G).

Isolated primary KCs from HS mice exhibited increased ferroptosis markers, such as MitoSOX, BODIPY 581/591 C11, FerroOrange, Liperfluo, and total iron, indicating enhanced ferroptosis under HS conditions (Fig. S2H–S2K).

In summary, our findings underscore the significant role of ferroptosis in KCs as a driver of liver injury in HS mice.

### 3.2 Single-cell RNA sequencing unveils distinct Kupffer cell subsets with variable ferroptosis susceptibility

**Figure 2:**
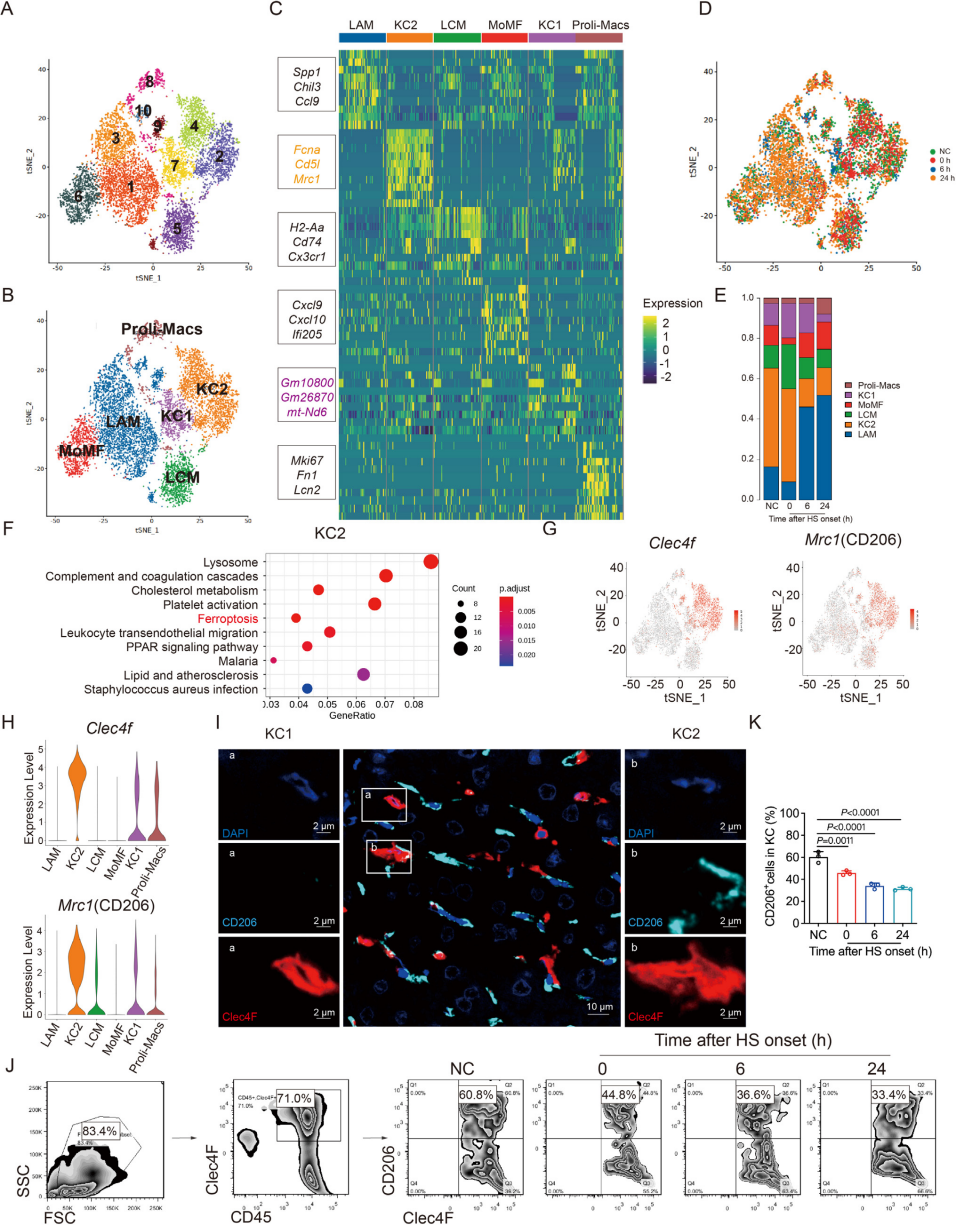
Unbiased approaches reveal macrophage heterogeneity with single-cell RNA sequencing. (A) tSNE plot displaying single-cell RNA sequencing data, with each color representing a distinct cluster. (B) tSNE plots illustrating cell subtype annotation. (C) Key marker genes for the respective cell subtypes. (D) tSNE plot showing the cell status of each sample. (E) Histogram depicting the proportion of cell subtypes in four sample groups: NC, HS at 0, 6, and 24 h. (F) KEGG pathway analysis was specific to the KC2 subtype. (G) tSNE graph and (H) violin graph displaying *Clec4f* and *Mrc1* (CD206) expression in all macrophage subclusters. (I) Immunofluorescence technology monitoring the expression of Clec4F and CD206 in KCs of normal mice. Clec4F^+^/CD206^–^ cells labeled as “a” belong to KC1, while Clec4F^+^/CD206^+^ cells labeled as “b” belong to KC2 (scale bar: 10 μm). (J) Flow cytometry monitoring the frequency of CD206^+^ KCs in HS mice recovered at 0, 6, and 24 h. (K) Statistical results (*n* = 3 mice per group) for CD206^+^ KC frequency. Data are presented as the mean ± SEM, and statistical significance was determined by one-way ANOVA with Tukey's test.

To unravel subsets of KCs with differing susceptibility to ferroptosis, we employed single-cell RNA sequencing on liver macrophages and monocytes from distinct groups: normal controls (NC) and HS at 0, 6, or 24 h (Supporting Information Fig. S3A). Our analysis focused on macrophages, excluding monocytes using classical markers such as *Lyz2*, *Sell*, *Ccr2*, and *Ly6c2*. Seurat analysis identified 10 main clusters, with differentially expressed genes (DEGs) delineating six distinct cell types (Fig. 2A and B).

Clusters 1, 3, 9, and 10 were annotated as lipid-associated macrophages (LAM) due to the expression of canonical population markers: *Trem2, Spp1, Ccl9,* and *Chil3*^30^. Cluster 5 was characterized as liver capsule macrophages (LCM), with high expression of *Cx3cr1* and MHC class II molecules *H2-Eb1/H2-Aa*^31^. Cluster 6 represented monocyte-derived macrophages (MoMF), exhibiting high expression of genes such as *Cxcl9, Cxcl10, Ifi205, Isg15,* and *Mx1*, akin to monocytes^32^. The expression of *Mki67, Fn1,* and *Lcn2* in Cluster 8 indicated a cluster of proliferative macrophages (Proli-Macs) (Fig. 2C).

Cluster 2, Cluster 4, and Cluster 7 were identified as KCs based on canonical population markers: *Cd5l, Vsig4, Fcna,* and *Clec4f*. Additionally, *Cd163* and *Mrc1* (CD206) were highly expressed in Cluster 2 and Cluster 4, while they exhibited low expression in Cluster 7. Therefore, Cluster 2 and Cluster 4 were annotated as KC2 (Clec4F^+^/CD206^+^KCs) and Cluster 7 as KC1 (Clec4F^+^/CD206^–^KCs)^33^. Notably, KC2 showed a significant reduction after 6 h of HS, indicating substantial KC2 death, while KC1 exhibited minimal changes (Fig. 2D, E, G, H).

KEGG pathway analysis revealed that KC2 was significantly associated with lysosome and ferroptosis pathways, distinguishing it from other macrophage subsets (Fig. 2F and Fig. S3B–S3F). Both KCs subtypes highly expressed the KCs marker *Clec4f*, with KC2 additionally expressing *Mrc1* (CD206) (Fig. 2I). Flow cytometry confirmed a reduction in KC2 numbers in HS mice, consistent with single-cell RNA sequencing results (Fig. 2J and K).

**Figure 3:**
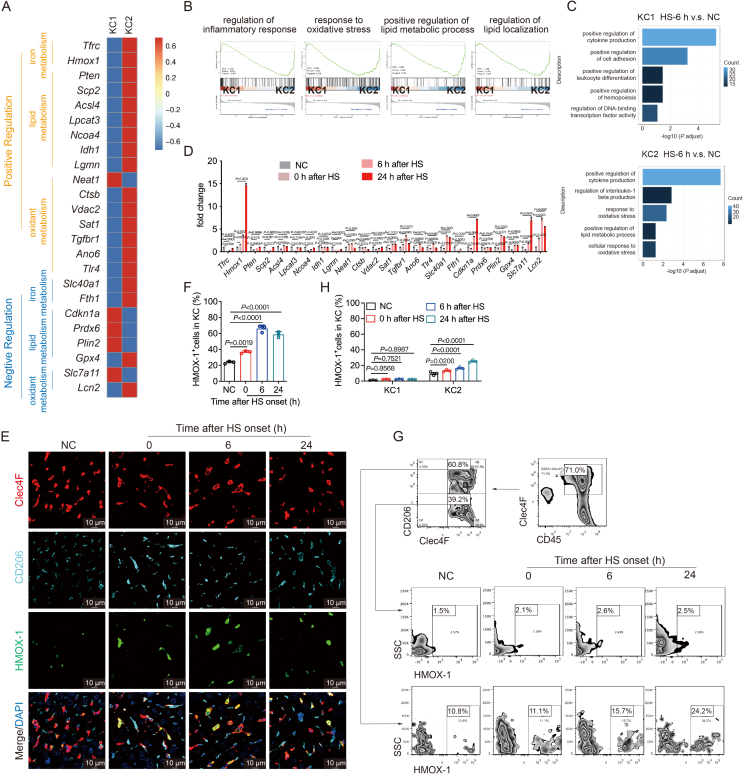
KC2 shows distinct susceptibility to ferroptosis. (A) Heat maps illustrate the expression of genes related to iron metabolism, lipid metabolism, and oxidant metabolism in KC1 and KC2. (B) Gene Set Enrichment Analysis (GSEA) plot showing the enrichment of gene sets in KC1 and KC2. (C) Gene Ontology (GO) pathway analysis of KC1 and KC2 from NC and HS-recovered mice at 6 h. (D) Measurement of relative mRNA levels of genes from (A) in KC2 subjected to heat stress (*n* = 4). (E) Representative images showing Clec4F (red), CD206 (cyan blue), and HMOX-1 (green) in liver paraffin sections of HS mice (scale bar: 10 μm), with statistical results (*n* = 3) (F). (G)Flow cytometry was used to detect HMOX-1 expression in Clec4F^+^/CD206^+^ and Clec4F^+^/CD206^–^ cells in liver tissues, with statistical analysis shown in (*n* = 3 mice per group) (H). Data are presented as the mean ± SEM, and statistical significance was determined using a one-way ANOVA with Tukey's *post hoc* test.

The analysis of ferroptosis-related genes related to iron and lipid metabolism, as well as oxidant processes, showcased distinct functions for KC1 and KC2 (Fig. 3A). Gene Set Enrichment Analysis (GSEA) highlighted KC2's significant enrichment in genes associated with inflammatory response, oxidative stress, and lipid metabolism (Fig. 3B). Gene Ontology (GO) analysis emphasized oxidative stress, lipid metabolism, and IL-1*β* regulation in KC2, while KC1 enriched in signaling pathways related to complement and coagulation cascades, cell adhesion, leukocyte differentiation and leukocyte migration (Fig. 3C and Fig. S3G–S3I). Focusing on *Hmox1*, the most significantly altered gene in the HS group, we observed high expression in KC2, further supporting the pivotal role of KC2 in ferroptosis (Fig. 3D–H).

In summary, these findings underscore the susceptibility of KC2 to ferroptosis induction, highlighting its significant contribution to liver injury during HS.

### 3.3 HMOX-1-mediated ferroptosis promotes liver injury in heat stroke mice

**Figure 4:**
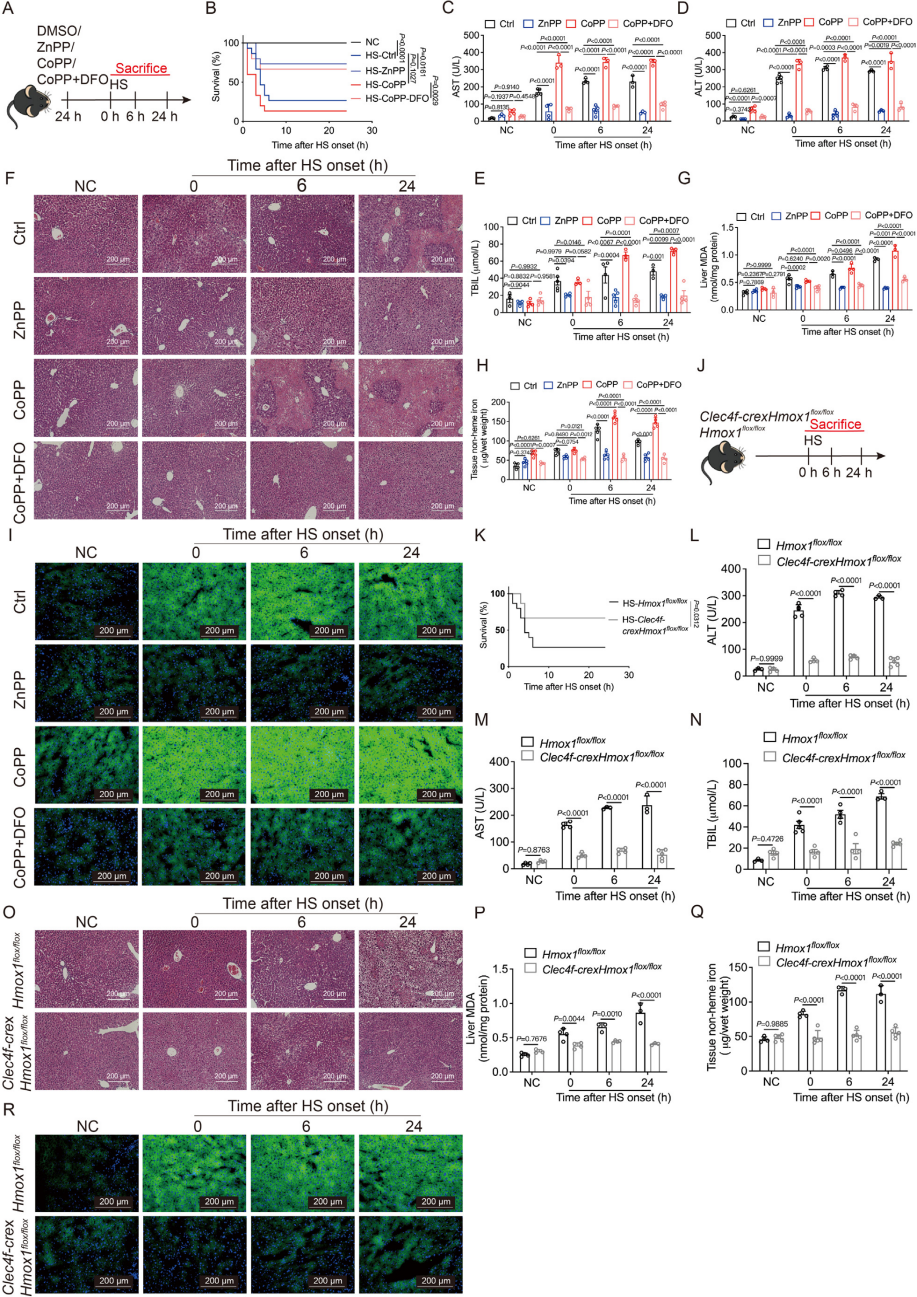
HMOX-1-specific targeting in KCs induces ferroptosis. (A) Schematic diagram illustrating the influence of HMOX-1 through ZnPP, CoPP, and CoPP + DFO. (B) Survival curves of mice pretreated with 10 mg/kg ZnPP, 5 mg/kg CoPP, or 5 mg/kg CoPP + 100 mg/kg DFO followed by HS (*n* = 15 mice per group). Evaluation of hepatocellular function by AST (C), ALT (D), and TBIL (E) (*n* = 3–5 mice per group). (F) Representative H&E staining of liver paraffin sections from HS mice (scale bar: 200 μm). Detection of MDA (G), tissue non-heme iron (H) (*n* = 3–5 mice per group), and BODIPY 581/591 C11 (I) in liver tissue of HS mice (scale bar: 200 μm). (J) Schematic diagram illustrating the influence of HMOX-1 through specific *Hmox1* knockout in KCs. (K) Survival curves of *Clec4f-crexHmox1*^*flox/flox*^ mice or *Hmox1*^*flox/flox*^ mice, followed by HS (*n* = 15 mice per group). Measurement of ALT (L), AST (M), and TBIL (N) (*n* = 3–5 mice per group). (O) Representative H&E staining of liver paraffin sections of *Clec4f-cre*x*Hmox1*^*flox/flox*^ mice or *Hmox1*^*flox/flox*^ mice (scale bar: 200 μm). Measurement of MDA (P), tissue non-heme iron (Q) (*n* = 3–5 mice per group), and BODIPY 581/591 C11 (R) in liver tissue (scale bar: 200 μm). Summary data are presented as the mean ± SEM. Significance was calculated using a one-way ANOVA with Tukey's *post hoc* test. Significance in (B) and (K) is determined by the log-rank (Mantel–Cox) test.

To robustly validate the role of HMOX-1 in KCs ferroptosis, we investigated whether alterations in HMOX-1 expression influenced ferroptosis in the livers of HS mice. Inhibition of HMOX-1 with zinc protoporphyrin (ZnPP) significantly reduced liver injury and increased the survival rate of mice, as illustrated in Fig. 4A–F. Conversely, the HMOX-1 agonist cobalt protoporphyrin (CoPP) had the opposite effect, and the ferroptosis inhibitor DFO counteracted the impact of CoPP. ZnPP treatment resulted in decreased levels of 4-hydroxynonenal (4-HNE), free iron, liver MDA, tissue non-heme iron, and lipid peroxidation in HS mice (Fig. 4G–I, Supporting Information Fig. S4A and S4B). Conversely, CoPP reversed these outcomes, and the ferroptosis inhibitor DFO nullified the effect of CoPP.

To establish a KCs-specific *Hmox1* knockout model, we generated *Hmox1*^*flox/flox*^ mice and crossed them with *Clec4f-cre*-tdTomato mice (Fig. S4C), resulting in mice with a KCs-specific knockout of *Hmox1*, referred to as *Clec4f-crexHmox1*^*flox/flox*^. In this model, we observed reduced liver injury and increased survival rates in HS mice (Fig. 4J–O), accompanied by a decrease in 4-HNE, free iron, liver MDA, and tissue non-heme iron (Fig. 4P–R, Fig. S4D and S4E).

Furthermore, isolated KC2, labeled with MitoSOX, BODIPY 581/591 C11, FerroOrange, and a lipid peroxidation probe Liperfluo, exhibited increased ferroptosis under heat treatment. ZnPP attenuated this effect, while CoPP exacerbated it, and Fer-1 counteracted the effect of CoPP (Supporting Information Fig. S5A–S5F). To further underscore the role of HMOX-1, stable KC cell lines (ImKC) with *Hmox1* knocked down and overexpressed were established using lentivirus (Fig. S5G). *Hmox1* knockdown reduced ImKC ferroptosis after heat treatment, whereas *Hmox1* overexpression increased cell ferroptosis, aligning with the effects of ZnPP and CoPP (Fig. S5H–S5L).

In summary, these comprehensive findings indicate that HMOX-1-mediated ferroptosis in KCs, especially KC2, induces liver injury under HS conditions. Modulation of HMOX-1 emerges as a potential therapeutic strategy for HS-related liver injury.

### 3.4 HMOX-1-dependent ferroptosis in Kupffer cells activates the NLRP3 inflammasome

**Figure 5:**
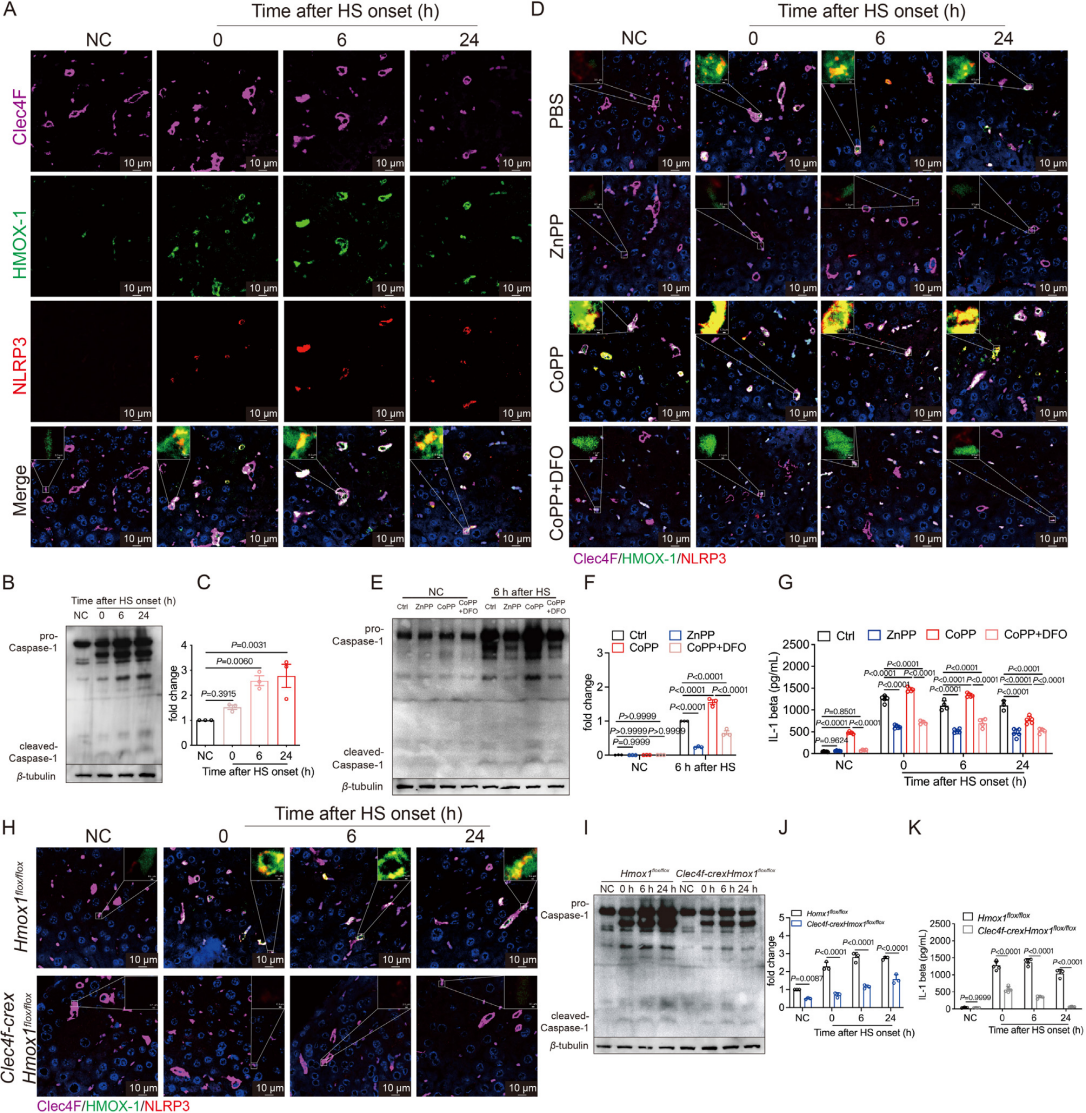
HMOX-1 affects NLRP3 inflammasome activation. (A) Representative immunofluorescence staining images displaying the expression of Clec4F (purple), HMOX-1 (green), NLRP3 (red), and in the liver of mice after HS, with magnified insets (scale bar: 10 μm). (B) Western Blotting analysis of Caspase-1 in liver tissues of HS mice and statistical results (*n* = 3) (C). (D) Representative images of immunofluorescence staining for Clec4F (purple), HMOX-1 (green), and NLRP3 (red) in liver tissues of HS mice pretreated with PBS, 10 mg/kg ZnPP, 5 mg/kg CoPP, or 5 mg/kg CoPP + 100 mg/kg DFO, with magnified insets (scale bar: 10 μm). (E) estern blotting analysis of caspase-1 in liver tissues of HS mice and statistical results (*n* = 3) (F). (G) Measurement of plasma IL-1*β* content by ELISA (*n* = 3–5 mice per group). (H) Representative images of immunofluorescence staining for Clec4F (purple), HMOX-1 (green), and NLRP3 (red) in liver tissues of *Clec4f-cre*x*Hmox1*^*flox/flox*^ mice or *Hmox1*^*flox/flox*^ mice, with magnified insets (scale bar: 10 μm). (I) Western blotting analysis of caspase-1 in liver tissues and statistical results (*n* = 3) (J). (K) Measurement of plasma IL-1*β* content by ELISA (*n* = 3–5 mice per group). Significance was calculated using a one-way ANOVA with Tukey's *post hoc* test.

Following the activation of the NLRP3 inflammasome, caspase-1 becomes activated and is responsible for the maturation and release of IL-1*β*^34^. Multicolor fluorescence staining of NLRP3 and IL-1*β* in the livers of HS mice revealed increased expression in KCs, co-localizing with HMOX-1 (Fig. 5A, Supporting Information Fig. S6A). Western Blotting further confirmed an elevation in cleaved caspase-1 in HS livers (Fig. 5B and C). The activation of NLRP3 in KCs decreased with ZnPP, while CoPP had the opposite effect. Co-administration of CoPP with DFO reversed the CoPP-induced effects (Fig. 5D, Fig. S6B). Elevated levels of cleaved caspase-1 in the liver and IL-1*β* in plasma were also detected in HS mice (Fig. 5E–G).

The specific knockout of *Hmox1* in KCs in *Clec4f-crexHmox1*^*flox/flox*^ mice resulted in decreased NLRP3 activation, IL-1*β* expression in KCs, and reduced cleaved caspase-1 (Fig. 5H–K, Fig. S6C). *Nlrp3* knockout reversed the effects of CoPP (Fig. S6D–S6H). Injection of ferroptosis inhibitors VE and liproxstatin-1 reduced cleaved caspase-1 production and IL-1*β* secretion (Fig. S6I–S6K).

In the KC2 cell experiment, an increased co-localization of NLRP3 with ASC was observed (Supporting Information Fig. S7A and S7B), indicating that heat treatment promotes the polymerization of NLRP3. To explore the relationship between NLRP3 activation and the trans-Golgi under heat treatment conditions, we stained NLRP3 and the trans-Golgi marker TGN38 in heat-treated primary KC2. We observed an increase in the co-localization of NLRP3 and TGN38 after heat treatment, and this effect was consistent with the results *in vivo* under ZnPP and CoPP treatments (Fig. S7C–S7F). Knocking down or overexpressing *Hmox1* in ImKCs yielded consistent results (Fig. S7G and S7H). Western blotting revealed that ZnPP or *sh-Hmox1* reduced cleaved caspase-1 production, while CoPP or *OE-Hmox1* had the opposite effect, paralleling the detection of plasma IL-1*β* (Fig. S7I–S7N).

In a co-culture model of KCs and hepatocytes, KC2 extracted from *Nlrp3*-knockout mice showed decreased hepatocyte death compared to WT KC2, indicating that NLRP3 inflammasome activation in KC2 directly affected hepatocyte death (Fig. S7O). Additionally, knockdown or inhibition of HMOX-1 in KCs reduced cell death, while overexpression or activation of HMOX-1 had the opposite effect (Fig. S7P and S7Q).

In summary, these comprehensive findings underscore that HMOX-1-dependent ferroptosis in KCs activates the NLRP3 inflammasome, contributing to the progression of liver injury under HS conditions.

### 3.5 PI4Kβ orchestrates PI4P aggregation on TGN, activating NLRP3

**Figure 6:**
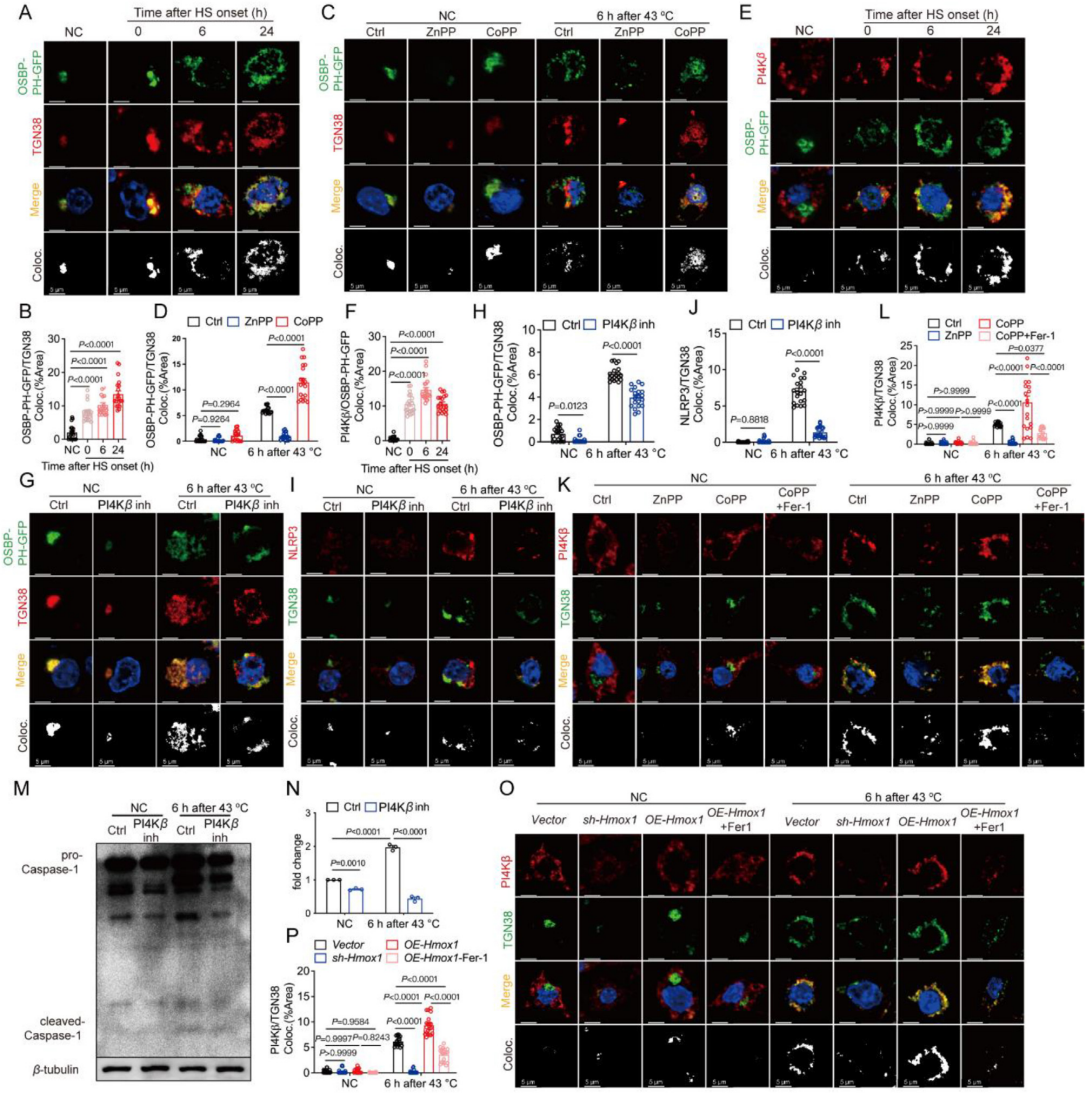
HMOX-1 affects PI4K*β* activation *in vitro*. (A) Representative images of immunofluorescence staining for OSBP-PH-GFP (green), TGN38 (red), and DAPI (blue) in KC2 treated at 43 °C for 3 h and recovered at 37 °C for 0, 6, or 24 h (scale bar: 5 μm), and statistical analysis (B) of co-localization of OSBP-PH-GFP and TGN38 (*n* = 20). (C) Representative images of immunofluorescence staining for OSBP-PH-GFP (green), TGN38 (red), and DAPI (blue) in KC2 pretreated with 4 μmol/L ZnPP for 12 h or 4 μmol/L CoPP for 12 h (scale bar: 5 μm), and statistical analysis (D) of co-localization of OSBP-PH-GFP and TGN38 (*n* = 20). (E) Representative images of immunofluorescence staining for OSBP-PH-GFP (green), PI4K*β* (red), and DAPI (blue) in KC2 (scale bar: 5 μm), and statistical analysis (F) of co-localization of OSBP-PH-GFP and PI4K*β* (*n* = 20). (G) Representative images of immunofluorescence staining for OSBP-PH-GFP (green), TGN38 (red), and DAPI (blue) in KC2 pretreated with 5 μmol/L PI4K*β* inhibitor for 1 h (scale bar: 5 μm), and statistical analysis (H) of co-localization of OSBP-PH-GFP and TGN38 (*n* = 20). (I) Representative images of immunofluorescence staining for NLRP3 (red), TGN38 (green), and DAPI (blue) in KC2 (scale bar: 5 μm), and statistical analysis (J) of co-localization of OSBP-PH-GFP and TGN38 (*n* = 20). (K) Representative images of immunofluorescence staining for PI4K*β* (red), TGN38 (green), and DAPI (blue) in KC2 pretreated with 4 μmol/L ZnPP for 12 h, 4 μmol/L CoPP for 12 h or 4 μmol/L CoPP for 12 h + 1 μmol/L Fer-1 for 1 h (scale bar: 5 μm), and statistical analysis (L) of co-localization of PI4K*β* and TGN38 (*n* = 20). (M) Western blotting analysis of caspase-1 in KC2 pretreated with DMSO or PI4K*β* inhibitor and (N) statistical results (*n* = 3). (O) Representative images of immunofluorescence staining for PI4K*β* (red), TGN38 (green), and DAPI (blue) in *vector*, *sh-Hmox1* or *OE-Hmox1* treated with 43 °C for 3 h and recovered at 37 °C for 6 h (scale bar: 5 μm) and (P) statistical analysis of co-localization of PI4K*β* and TGN38 (*n* = 20). Summary data are presented as the mean ± SEM. Significance was calculated using a one-way ANOVA with Tukey's *post hoc* test.

NLRP3 activation necessitates a scaffold containing a product of lipid metabolism phosphatidylinositol 4-phosphate (PI4P) on the trans-Golgi network (TGN)^35^. To delve into the involvement of PI4P in NLRP3 activation within KC2 post-heat treatment, we overexpressed the OSBP-PH-GFP (PI4P probe) in primary KC2. This revealed an augmented co-localization of PI4P with the trans-Golgi marker TGN38 and NLRP3 after heat treatment (Fig. 6A and B, Supporting Information Fig. S8A and S8B). ZnPP treatment in KC2 diminished PI4P-TGN38 co-localization, while CoPP heightened this co-localization (Fig. 6C and D). Consistently, *Hmox1* knockdown decreased PI4P-TGN38 co-localization, while *Hmox1* overexpression increased it (Fig. S8C and S8D). This implies that HMOX-1 regulates NLRP3 inflammasome activation in KC2 by influencing the lipid PI4P synthesis on TGN following heat treatment.

Further mechanistic exploration indicated that only the PI4K*β* inhibitor significantly reduced PI4P levels in KC2 (Fig. S8E). Concentration-dependent treatment with the PI4K*β* inhibitor decreased caspase-1 expression in heat-treated KC2 (Fig. S8F and S8G). PI4K*β* accumulated on TGN38 after heat treatment, co-localizing with PI4P and NLRP3 (Fig. 6E and F, Fig. S8H–S8K). Treatment with the PI4K*β* inhibitor reduced PI4P levels on TGN38, NLRP3 aggregation on TGN38, and cleaved caspase-1 expression in heat-treated KC2 (Fig. 6G–J, M, N, Fig. S8L and S8M). Knockdown of *PI4Kβ* in KC2 produced analogous results (Fig. S8N–S8V).

Intriguingly, the HMOX-1 inhibitor ZnPP attenuated PI4Kβ-TGN38 aggregation, while CoPP amplified it, with Fer-1 counteracting the effect of CoPP (Fig. 6K and L). *Hmox1* knockdown reduced PI4K*β*-TGN38 co-localization, while *Hmox1* overexpression increased it, and Fer-1 nullified the effect of *Hmox1* overexpression (Fig. 6O and P).

In summary, our findings underscore that HMOX-1 facilitates NLRP3 activation by promoting PI4K*β* aggregation on TGN38, ultimately leading to PI4P production in KC2 following heat treatment.

### 3.6 EGR1 orchestrates Hmox1 transcription

**Figure 7:**
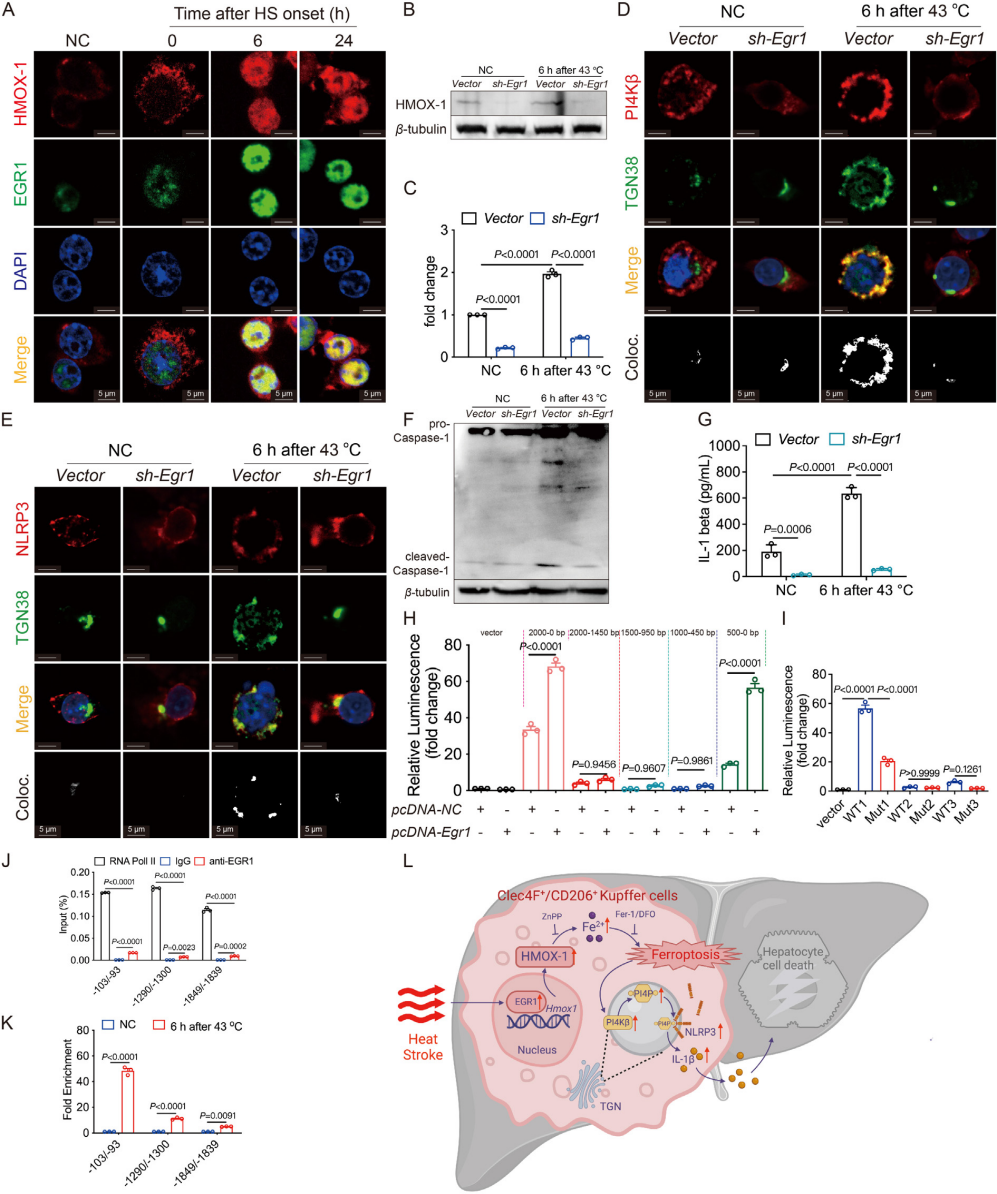
EGR1 regulates the transcription of *Hmox1*. (A) Representative images of immunofluorescence staining for HMOX-1 (red), EGR1 (green), and DAPI (blue) in KC2 treated at 43 °C for 3 h and recovered at 37 °C for 0, 6, or 24 h (scale bar: 5 μm). (B) Western blotting analysis of the expression of HMOX-1 after knocking down *Egr1* (*sh-Egr1*) in ImKCs and statistical analysis (*n* = 3) (C). (D) Representative images of immunofluorescence staining for PI4K*β* (red), TGN38 (green), and DAPI (blue) in *vector* or *sh-Egr1* (scale bar: 5 μm). (E) Representative images of immunofluorescence staining for NLRP3 (red), TGN38 (green) and DAPI (blue) (scale bar: 5 μm). (F) Western blotting analysis of caspase-1. (G) Cellular supernatant IL-1*β* content detected by ELISA (*n* = 3 mice per group). (H) Dual-luciferase experiments in HEK-293T cells transfected with different truncated regions of the *Hmox1* promoter (*n* = 3). (I) Dual-luciferase experiments in HEK-293T cells transfected with different mutational regions of the *Hmox1* promoter (*n* = 3). ChIP-qRT-PCR analysis of EGR1 binding to −103 to −93, −1300 to −1290, −1849 to −1839 sites in the *Hmox1* promoter region (*n* = 3) (J) and fold change of signals in ImKC treated with 43 °C for 3 h and recovered at 37 °C for 6 h (*n* = 3) (K). (L) A hypothetical model for KC2 ferroptosis in HS mice. Summary data are presented as the mean ± SEM. Significance was calculated using a one-way ANOVA with Tukey's *post hoc* test.

To decipher the transcriptional regulation of HMOX-1, we utilized PROMO and the GeneCards database to predict potential transcription factors, identifying EGR1 as a candidate. In single-cell RNA sequencing, *Egr1* exhibited up-regulation in HS KC2 (Supporting Information Fig. S9A), and immunofluorescence staining corroborated an increase in EGR1 expression and its nuclear translocation upon heat treatment (Fig. 7A).

To unravel EGR1's impact on *Hmox1* regulation, ImKCs with *Egr1* knockdown were generated, resulting in diminished HMOX-1 expression (Fig. 7B and C, Fig. S9B and S9C). NLRP3 inflammasome activation was adversely affected, marked by reduced co-localization of PI4K*β*, PI4P, and NLRP3 on TGN38 (Fig. 7D and E and Fig. S9D–S9G). This, in turn, led to diminished NLRP3 activation, cleaved caspase-1 expression, IL-1*β* secretion, and cell death (Fig. 7F and G, Fig. S9H).

Predicting EGR1's binding regions on the *Hmox1* promoter, we confirmed its primary interaction with the first predicted binding site (region 1) through Duo-Luciferase and ChIP-qRT-PCR experiments (Fig. 7H–K, Fig. S9I).

In summary, EGR1 emerges as a pivotal regulator of HMOX-1 expression in KC2, orchestrating ferroptosis and NLRP3 inflammasome activation, thereby contributing to liver injury in the context of heat stroke conditions (Fig. 7L).

## 4 Discussion

Distinct from sepsis-induced liver damage, HS-induced liver injury unfolds as a consequence of sterile inflammation, arising not from biochemical molecules but rather from the physical factor of heat. Cellular heat cytotoxicity in HS exposes the liver's early vulnerability to temperature-induced damage, initiating a complex interplay between thermocytotoxicity and the systemic inflammatory response syndrome (SIRS)^36^^,^^37^.

The general clinical manifestations of HS-related liver injury encompass a range of symptoms, including abdominal pain, nausea, vomiting, and fatigue. These symptoms often manifest in patients with early liver injury, where serum levels of liver enzymes such as ALT, AST, and TBIL may show a significant increase within the first 24 h of hospital admission^38^. Histological examination of liver tissue from individuals with HS-related liver injury may reveal hepatocyte necrosis, infiltration of inflammatory cells, and evidence of oxidative stress^1^^,^^28^. These findings collectively underscore the multifaceted nature of liver injury in the context of HS, highlighting the importance of early recognition and intervention to mitigate potential complications.

Elevated levels of ALT, AST, and TBIL serve as markers of severe liver damage, contributing directly to patient mortality^38^^,^^39^. Bilirubin elevation, primarily originating from the breakdown of hemoglobin in KCs, signifies liver dysfunction, underscoring the intricate role of KCs in HS-related liver injury^40^^,^^41^. KCs, positioned at the epicenter of inflammation regulation, become key contributors to HS-related liver damage by secreting pro-inflammatory mediators^42^, ^43^, ^44^, ^45^. Our findings unveil a significant elevation of IL-1*β* in HS mice, with a subsequent decrease after KC depletion. IL-1*β* appeared at HS recovery 0 h, while monocyte-macrophages appeared at 6 h or even later. Additionally, neutrophils were also detected at 6 h (data not shown). This temporal sequence highlights KCs’ early response, preceding monocyte-derived macrophages, and neutrophils, in the intricate orchestration of the inflammatory cascade during HS recovery. Such insights carry profound therapeutic implications for effectively managing HS-related inflammation.

Our results align with previous research indicating that M2 macrophages, with characteristics similar to KC2, are more susceptible to ferroptosis because NO· in M1 macrophages suppresses ferroptosis in macrophages^46^. HMOX-1 (also known as heat shock protein 32, which is sensitive to heat), a key enzyme responsible for biliverdin conversion in the liver, emerges as a crucial player in this process. The role of HMOX-1 in antioxidant defense and its potential to promote ferroptosis is a matter of ongoing debate, with expression levels likely playing a crucial role in this context. In conditions where HMOX-1 expression is moderately increased (generally less than 5-fold), it can prevent oxidative stress and exhibit a protective function in the liver with ischemia-reperfusion^47^^,^^48^. On the contrary, excessive up-regulation of HMOX-1 (usually exceeding 15-fold) can lead to the generation of large amounts of reactive iron, ultimately inducing cell death^23^^,^^49^. Contrary to its recognized role in antioxidant defense, it has been reported that HMOX-1 promotes ferroptosis of hepatic stellate cells and thus affects the progression of liver fibrosis^50^. Studies have also shown that HMOX-1 promotes ferroptosis in hepatocellular carcinoma^51^. In our study, HMOX-1's excessive expression in KCs, particularly in the identified KC2 subtype, promotes ferroptosis, exacerbating liver injury in HS mice. Targeting HMOX-1 in KCs presents a potential therapeutic avenue for mitigating HS-related inflammation.

The NLRP3 inflammasome activation serves as the linchpin for the maturation and release of IL-1*β*. Dysregulation of the NLRP3 inflammasome contributes to various diseases associated with ferroptosis, and there is evidence suggesting that GPX4 inhibits its activation^52^^,^^53^. Additionally, Xiang's research group^54^ investigated in a hindlimb ischemia model, high HMOX-1 expression promoted NLRP3 inflammasome activation, worsening inflammation, and vascular injury. The activation of NLRP3 requires a scaffold on the TGN with PI4P^35^. While the study indicates that PI4P promotes lipid peroxidation in macrophages, leading to IL-1*β* secretion, the focus of that research was on pyroptosis in bone marrow-derived macrophages within a sepsis model^55^. Nevertheless, the observed phenomenon of lipid peroxidation promoting macrophage death and activating IL-1*β* secretion provides valuable insights. PI4K*β* is commonly targeted to inhibit the proliferation of viruses or parasites^56^^,^^57^. Our study revealed PI4K*β*′s role in NLRP3 activation during HS, with HMOX-1 affecting PI4K*β* aggregation on the TGN. Further research is needed to elucidate how HMOX-1 influences PI4K*β* on the TGN.

EGR1 responds to various stressors, including cytokines and oxidative stress^58^, ^59^, ^60^. Dennery's group^61^ found homology between the *Hmox1* promoter and EGR1 binding region. Although EGR1 is recognized in M2 macrophage polarization, its role in IL-1*β* regulation is less explored^62^, ^63^, ^64^. EGR1's role in IL-1*β* regulation, particularly in the context of HS, signifies its potential as a therapeutic target.

## 5 Conclusions

This comprehensive study elucidates the intricate molecular mechanisms underpinning HS-induced liver injury, highlighting the diverse responses of KCs subsets and the pivotal role of HMOX-1 in orchestrating ferroptosis in KCs, particularly in KC2, and NLRP3 inflammasome activation. Targeting specific subsets of KCs and modulating key molecular players like HMOX-1 and PI4K*β* emerges as promising strategies for addressing the complex pathophysiology of HS-related conditions.

## Acknowledgments

This work was supported by the following funding sources: the 10.13039/501100001809National Natural Science Foundation of China (82072100 to Qiang Ma and 82172814 to Liying Zhao), the Natural Science Foundation of Shenzhen (JCYJ20210324120212033, China) and Guangdong Provincial Key Laboratory of Immune Regulation and Immunotherapy, School of Laboratory Medicine and Biotechnology, Southern Medical University (2022B1212010009, China).

## Author contributions

Ru Li: Data curation, Methodology, Validation, Visualization, Writing – original draft. Riqing Wei: Conceptualization, Methodology, Validation, Visualization, Writing – review & editing. Chenxin Liu: Data curation, Methodology. Keying Zhang: Methodology, Visualization. Sixiao He: Software. Zhifeng Liu: Resources, Software. Junhao Huang: Software. Youyong Tang: Validation. Qiyuan An: Validation. Ligen Lin: Formal analysis. Lishe Gan: Formal analysis. Liying Zhao: Funding acquisition, Resources. Xiaoming Zou: Conceptualization, Supervision, Writing – review & editing. Fudi Wang: Conceptualization, Project administration, Writing – review & editing. Yuan Ping: Conceptualization, Project administration, Writing – review & editing. Qiang Ma: Conceptualization, Funding acquisition, Supervision, Writing – review & editing.

## Conflicts of interest

The authors declare no competing interests.

## Appendix A Supporting information

The following is the Supporting information to this article:
