# Supplementary material for: Heme oxygenase 1-mediated ferroptosis in Kupffer cells initiates liver injury during heat stroke
Source: Acta Pharm Sin B. 2024 May 13;14(9):3983–4000. doi: 10.1016/j.apsb.2024.05.007 (PMC11413699; doi:10.1016/j.apsb.2024.05.007)
Supplement: Multimedia component 1 [file mmc1.pdf]

Supporting Information for

Original article

Heme oxygenase 1-mediated ferroptosis in Kupffer cells initiates liver injury during heat stroke

Ru Li[a,b,†], Riqing Wei[b,†], Chenxin Liu[b], Keying Zhang[b], Sixiao He[b], Zhifeng Liu[c,d], Junhao Huang[b], Youyong Tang[b], Qiyuan An[b], Ligen Lin[e], Lishe Gan[f], Liying Zhao[g], Xiaoming Zou[a,*], Fudi Wang[h,i,*], Yuan Ping[j,k,*], Qiang Ma[b,l,*]

[a]*The Seventh Affiliated Hospital, Southern Medical University, Foshan 528244, China*

[b]*Department of Biopharmaceutics, School of Laboratory Medicine and Biotechnology, Southern Medical University, Guangzhou 510000, China*

[c]*Medical Critical Care Medicine, General Hospital of Southern Theatre Command of PLA, Guangzhou 510000, China*

[d]*Guangdong Branch Center, National Clinical Research Center for Geriatric Diseases (Chinese PLA General Hospital), Guangzhou 510000, China*

[e]*State Key Laboratory of Quality Research in Chinese Medicine, Institute of Chinese Medical Sciences, University of Macau, Avenida da Universidade, Taipa, Macao 999078, China*

[f]*School of Pharmaceutical Sciences, Zhejiang Chinese Medical University, Hangzhou 311402, China*

[g]*Department of General Surgery, Nanfang Hospital, the First School of Clinical Medicine, Southern Medical University, Guangzhou 510000, China*

[h]*The Fourth Affiliated Hospital, the First Affiliated Hospital, School of Public Health, Institute of Translational Medicine, Cancer Center, State Key Laboratory of Experimental Hematology, Zhejiang University School of Medicine, Hangzhou 310000, China*

[i]*The First Affiliated Hospital, the Second Affiliated Hospital, Basic Medical Sciences, School of Public Health, Hengyang Medical School, University of South China, Hengyang 421200, China*

[j]*College of Pharmaceutical Sciences, Zhejiang University, Hangzhou 310000, China*

[k]*Liangzhu Laboratory, Zhejiang University Medical Center, Hangzhou 310000, China*

[l]*Guangdong Provincial Key Laboratory of Immune Regulation and Immunotherapy, School of Laboratory Medicine and Biotechnology, Southern Medical University, Guangzhou 510000, China*

Received 25 December 2023; received in revised for 5 March 2024; accepted 15 March 2024

*Corresponding authors.

E-mail addresses: 1814925716@qq.com (Xiaoming Zou), : fwang@zju.edu.cn (Fudi Wang), pingy@zju.edu.cn (Yuan Ping), mq@smu.edu.cn (Qiang Ma).

†These authors made equal contributions to this work.

1. Supporting Tables

Table S1 Characteristics of individuals with HS included in the study.

Characteristic	Mean ± SEM	Minimum value	Maximum value
Age	26.94±2.067	17	66
ALT (U/L)	479.1±152	15	2868
AST (U/L)	571.3±158.2	19	2892
TBIL (μmol/L)	36.28±7.536	9.4	169.8

Table S2 Characteristics of healthy control included in the study.

Characteristic	Mean ± SEM	Minimum value	Maximum value
Age	35.66±1.32	21	57
ALT (U/L)	24.88±2.919	6	78
AST (U/L)	22.84±1.513	9	54
TBIL (μmol/L)	11.72±0.948	5.5	28.8

2. Supporting Figures

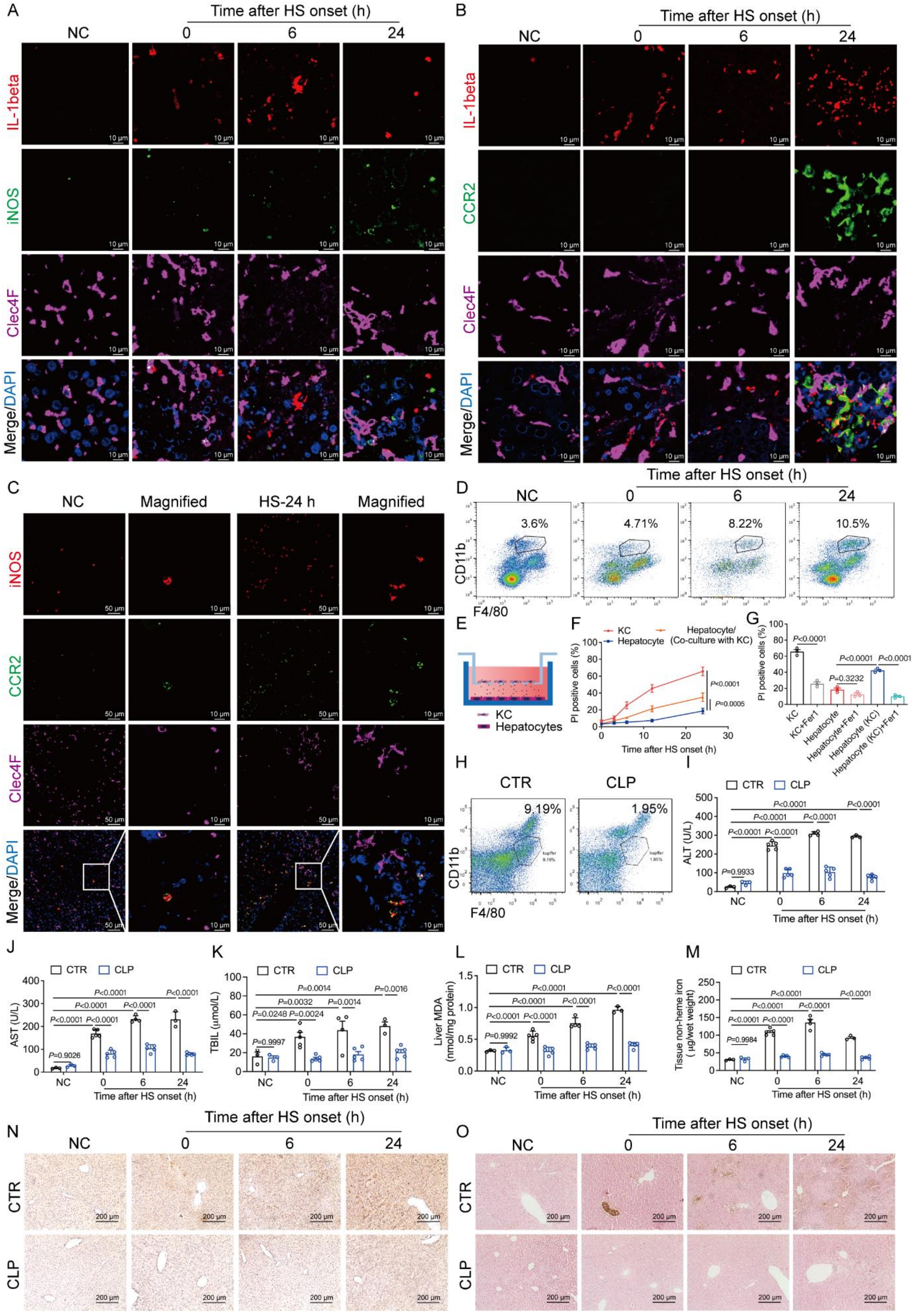

Figure S1. KCs exacerbated liver damage during HS onset. (**A–C**) Representative immunofluorescence staining images displaying the expression of IL-1 beta, iNOS, CCR2, and Clec4F in the liver of mice after HS (scale bar: 10 μm). (**D**) Flow cytometry analysis of monocyte-macrophage changes in HS mice, labeled as F4/80^{low} and CD11b^{hi} cells. (**E**) Schematic of hepatocytes and KCs co-culture. (**F, G**) Cell death assessment (PI-positive) in hepatocytes, KCs, and co-cultured cells after heat stress, with or without Fer-1 pretreatment (n=3). (**H**) Flow cytometry analysis of KC changes in mice after CLP treatment, labeled as F4/80^{hi} and CD11b^{low} cells. Evaluation of hepatocellular function using ALT (**I**), AST (**J**), TBIL (**K**), MDA (**L**) and non-heme iron content (**M**) in liver tissue (n=3–5 mice per group). (**N**) Representative immunohistochemical images of 4-HNE (scale bar: 200 μm) and (**O**) Perls' Prussian Blue staining in liver tissue from HS mice pretreated with CLP or CTR (scale bar: 200 μm). Data are presented as mean ± SEM. Significance was assessed using one-way ANOVA with Tukey's *post hoc* test.

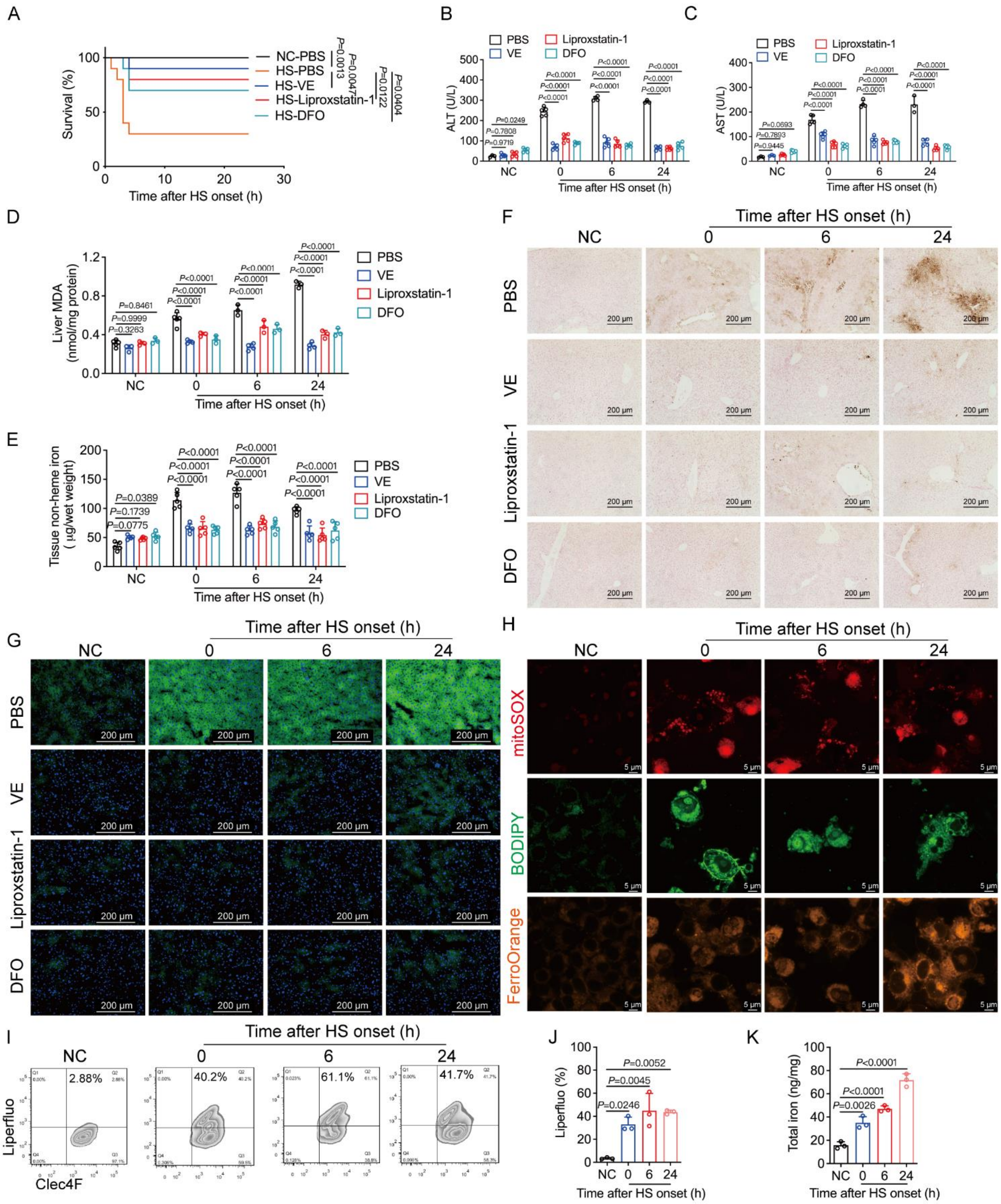

Figure S2. Contribution of ferroptosis in KCs to liver injury in HS mice. (**A**) Survival curves of mice pretreated with PBS, 500 mg/kg VE, 10 mg/kg Liproxstatin-1, or 100 mg/kg DFO followed by HS (*n*=10 mice per group). Assessment of hepatocellular function through ALT (**B**) and AST (**C**) in various experimental groups (*n*=3–5 mice per group). Quantification of MDA (**D**) and tissue non-heme

iron content (**E**) in liver tissue (*n*=3–5 mice per group). Representative staining images of Perls' Prussian Blue staining (**F**) and BODIPY 581/591 C11 staining (**G**) in liver tissue (scale bar: 200 μm). (**H**) Representative images of KCs stained for mitoSOX (mitochondrial ROS), BODIPY (lipid oxidation), and FerroOrange (Fe^{2+}) (scale bar: 200 μm). (**I**) Flow cytometry analysis of lipid peroxidation (Clec4F^{+}/Liperfluo^{+}) in KCs from HS mice, followed by statistical analysis (*n*=3 mice per group) (**J**). (**K**) Measurement of total iron in KCs of HS mice using a kit (*n*=3 mice per group). Data presented as mean ± SEM. Significance was assessed using one-way ANOVA with Tukey's *post hoc* test.

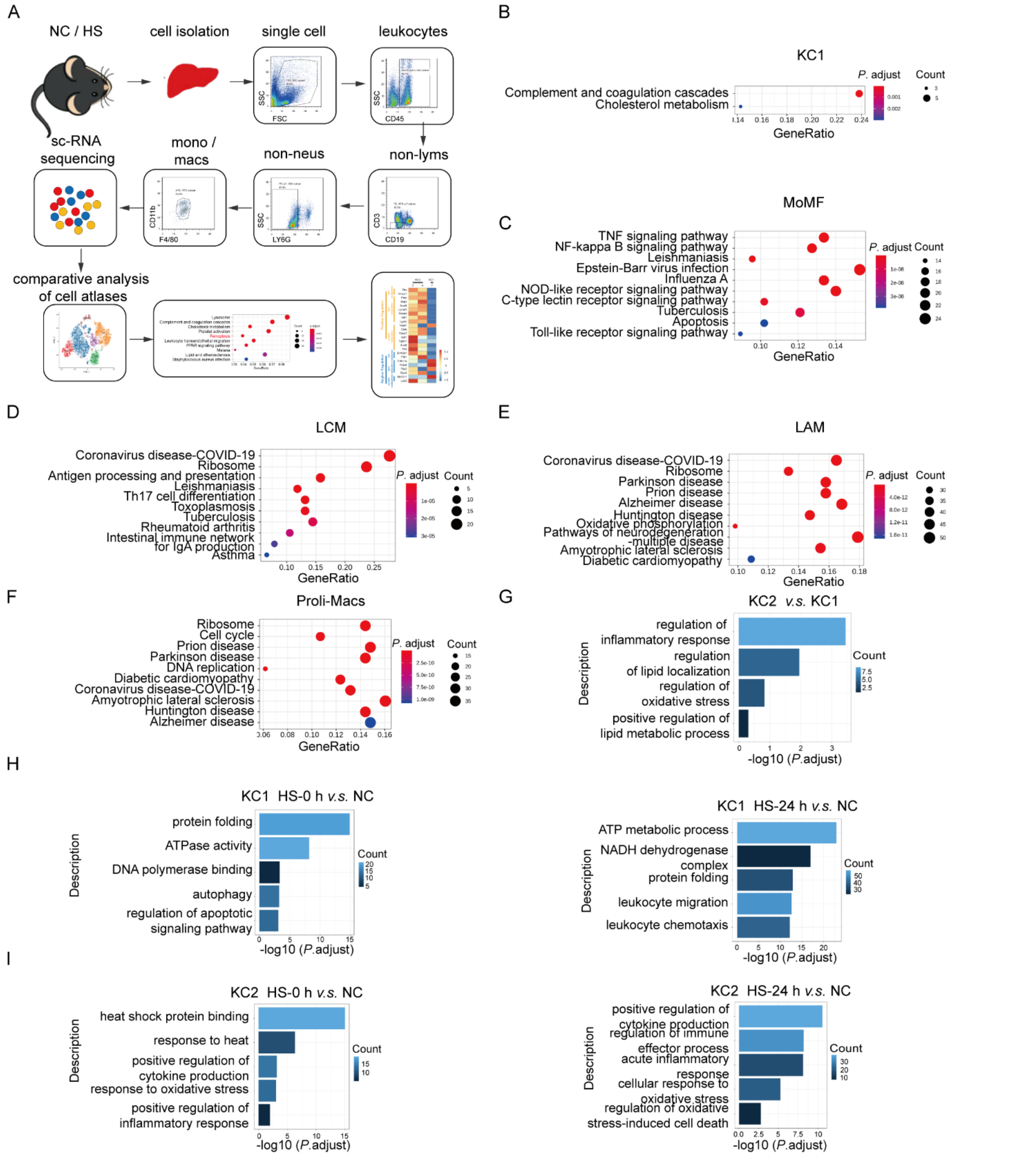

Figure S3. Functional analysis of macrophage subsets. (**A**) Flowchart illustrating the single-cell RNA sequencing experiment. (**B–F**) KEGG pathway analysis of distinct macrophage subsets: KC1 (**B**), MoMF (**C**), LCM (**D**), LAM (**E**), and Proli-Macs (**F**). (**G**) GO pathway analysis of KC2 *vs.* KC1. (**H**) GO pathway analysis of KC1 from NC and HS-recovered mice at 0 and 24 h. (**I**) GO pathway analysis of KC2 from NC and HS-recovered mice at 0 and 24 h.

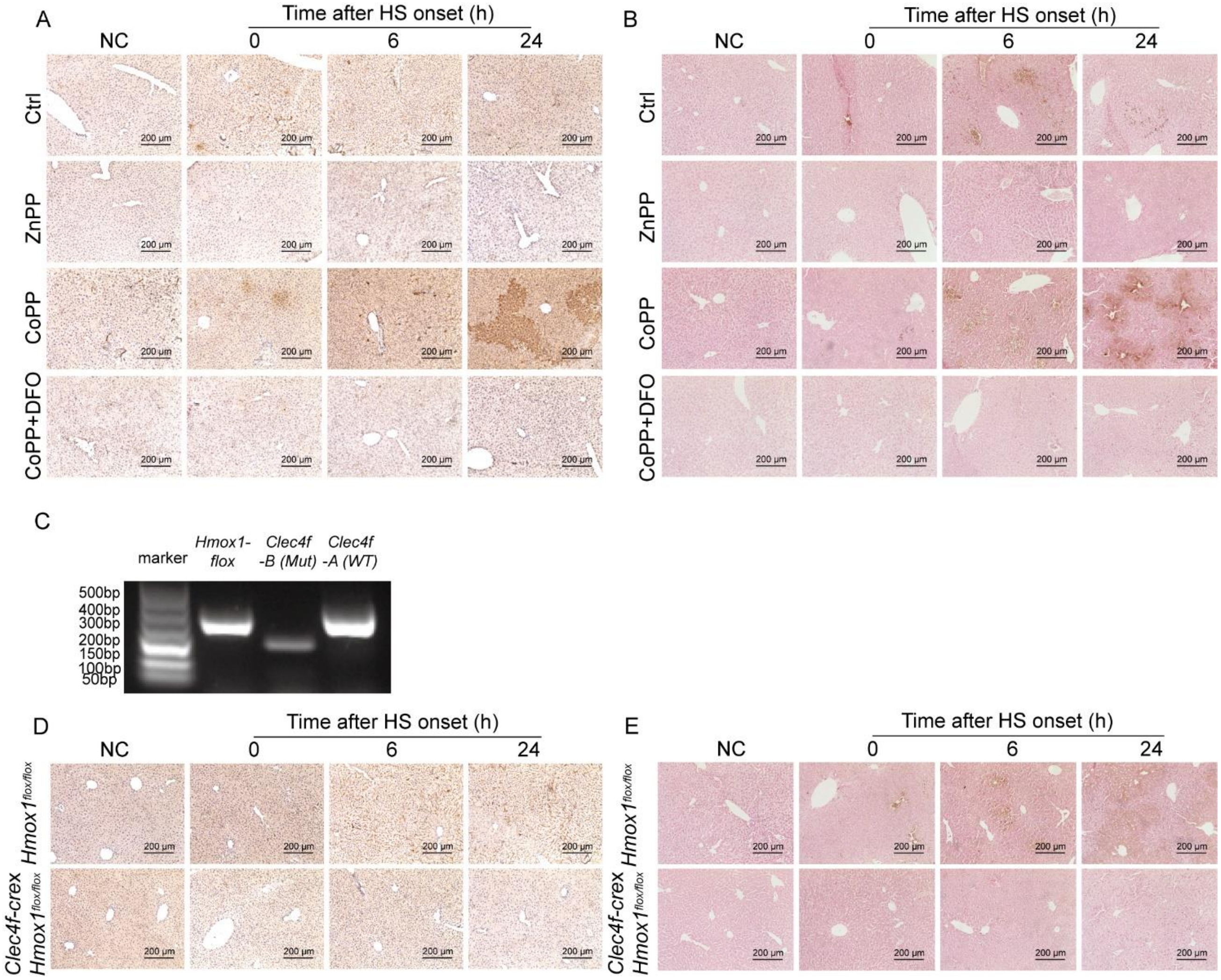

Figure S4. HMOX-1 induces ferroptosis in KCs. (**A**) Representative immunohistochemical staining images for 4-HNE in liver tissue from HS mice pretreated with DMSO, 10 mg/kg HMOX-1 inhibitor ZnPP, 5 mg/kg HMOX-1 agonist CoPP, or 5 mg/kg CoPP followed by 100 mg/kg ferroptosis inhibitor DFO (scale bar: 200 μm). (**B**) Representative images of Perls' Prussian Blue staining in HS mice liver sections (scale bar: 200 μm). (**C**) Genetic validation of *Clec4f-crexHmox1$^{flox/flox}$* mice. (**D**) Representative immunohistochemical staining for 4-HNE in liver tissue from *Clec4f-crexHmox1$^{flox/flox}$* mice and littermate *Hmox1$^{flox/flox}$* mice (scale bar: 200 μm). (**E**) Representative images of Perls' Prussian Blue staining in the livers of HS mice (scale bar: 200 μm).

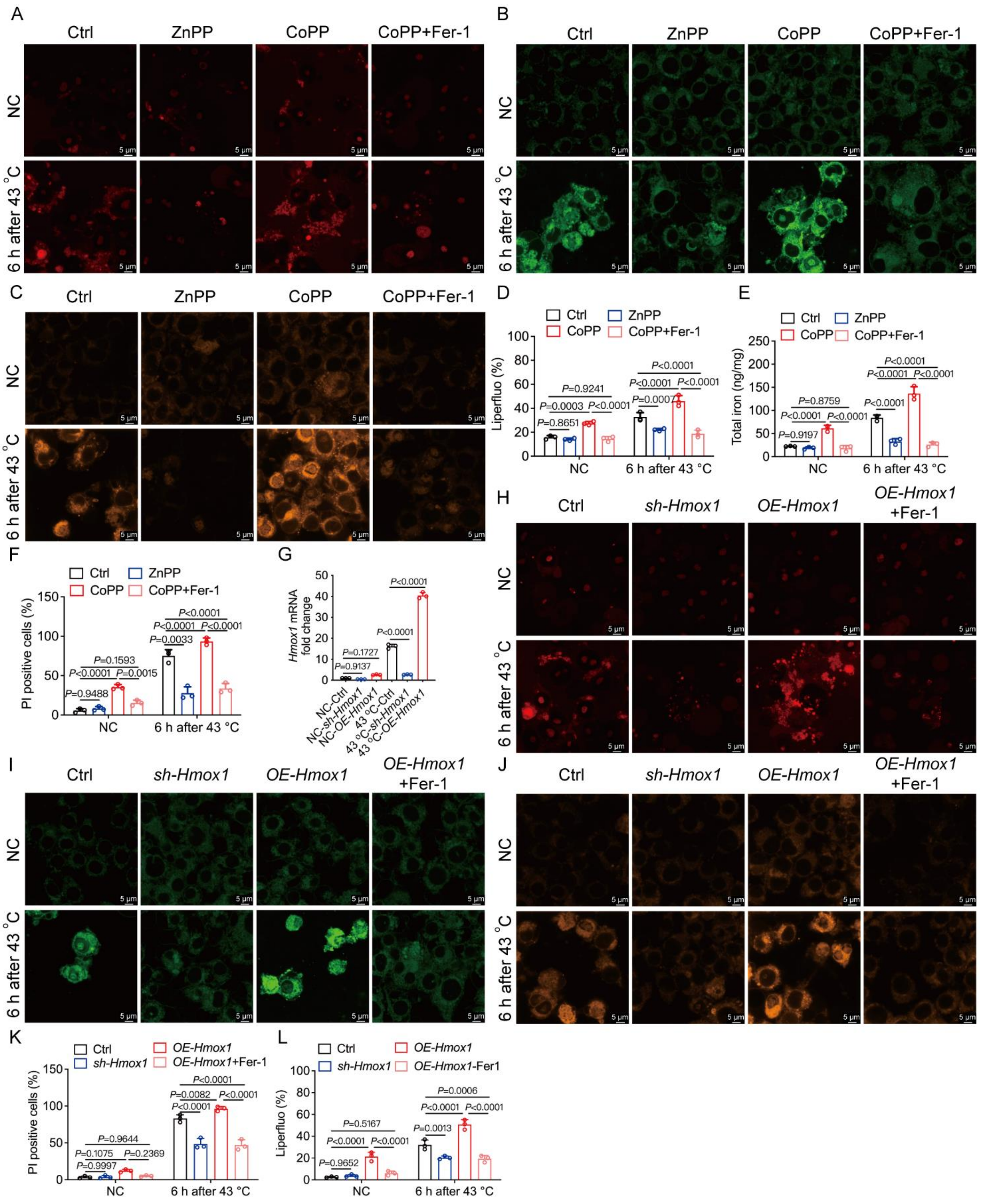

Figure S5. HMOX-1 induces ferroptosis in KC2. Representative staining images of KC2 labeled with mitoSOX (mito ROS) (**A**), BODIPY (lipid oxidation) (**B**), and FerroOrange (Fe^{2+}) (**C**) at 43 °C for 3 h, followed by recovery for 6 h after pretreatment with PBS, 4 μmol/L ZnPP, 4 μmol/L CoPP, or 4 μmol/L CoPP + 1 μmol/L Fer-1 (scale bar: 5 μm). (**D**) Analysis of lipid peroxidation (Liperfluo^{+}) in

KC2 using flow cytometry (*n*=3). Total iron (**E**) and cell death detection (PI positive) (**F**) of KC2 (*n*=3). (**G**) Relative mRNA levels of *Hmox1* in *sh-Hmox1* ImKC stable transfected cell line with *Hmox1* knockdown and *OE-Hmox1* ImKC stable transfected cell line with *Hmox1* overexpression after heat stress treatment (*n*=3). Representative staining images of ImKC labeled with mitoSOX (mito ROS) (**H**), BODIPY (lipid oxidation) (**I**), and FerroOrange (Fe^{2+}) (**J**) (scale bar: 5 μm). (**K**) Cell death detection (PI positive) of ImKC (*n*=3). (**L**) Lipid peroxidation (Liperfluo^{+}) in ImKC analyzed by flow cytometry (*n*=3). Data are presented as the mean ± SEM. Significance was determined using a one-way ANOVA with Tukey's *post hoc* test.

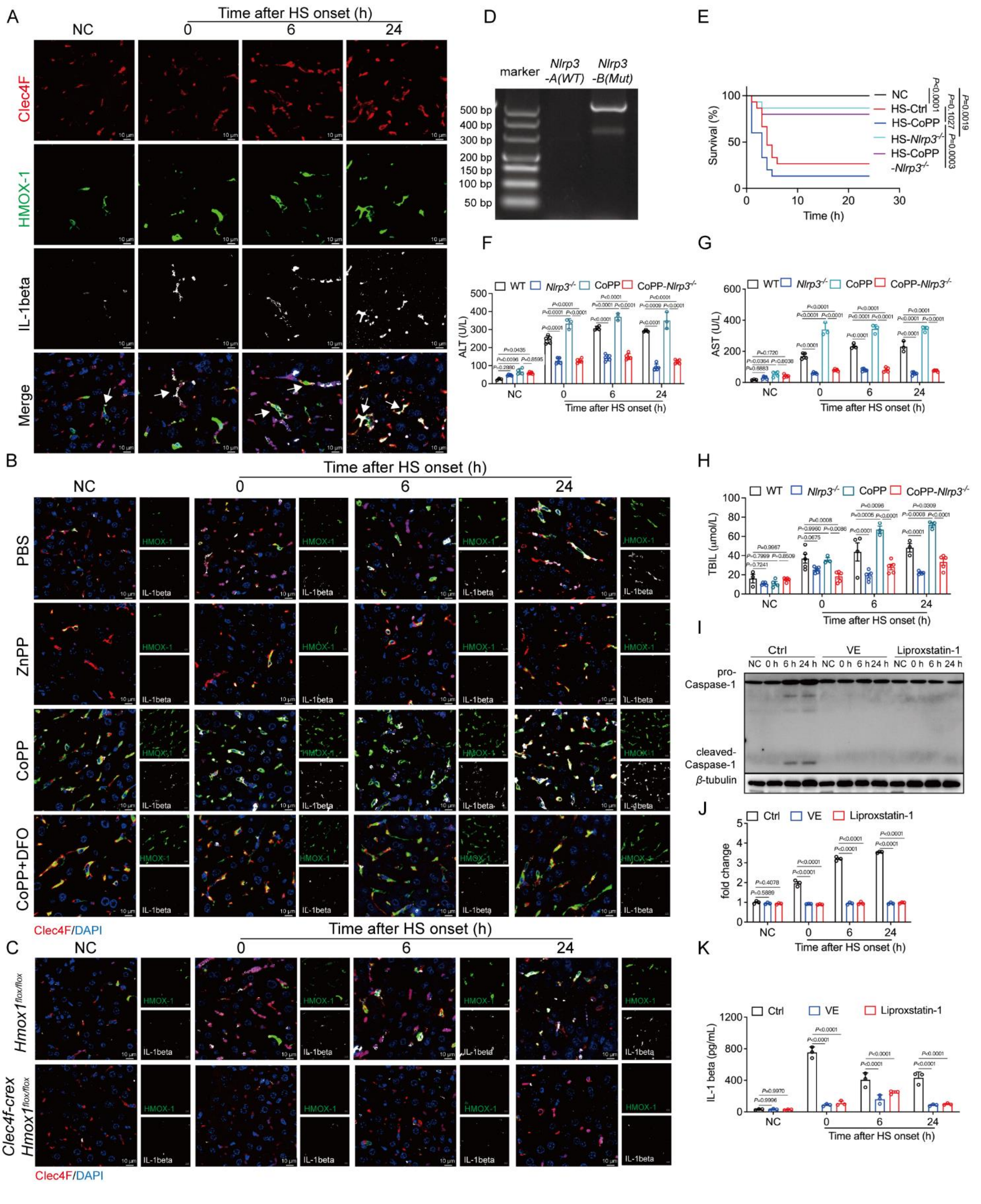

Figure S6. Inhibition of ferroptosis reduces NLRP3 inflammasome activation in HS mice. (**A**) Representative images of Clec4F (red), HMOX-1 (green), and IL-1β (white) in liver paraffin sections of HS mice (scale bar: 10 μm). (**B**) Representative images of immunofluorescence staining in liver tissues of HS mice pretreated with PBS, 10 mg/kg ZnPP, 5 mg/kg CoPP, or 5 mg/kg CoPP + 100 mg/kg

DFO (scale bar: 10 μm). (**C**) Representative images of immunofluorescence staining in liver tissues of *Clec4f-cre*x*Hmox1*$^{flox/flox}$ mice or *Hmox1*$^{flox/flox}$ mice (scale bar: 10 μm). (**D**) Genetic verification of *Nlrp3*$^{-/-}$ mice. (**E**) Survival curves of mice pretreated with CoPP for wild-type mice or *Nlrp3*$^{-/-}$ mice, followed by HS (n =15 mice per group). Evaluation of hepatocellular function by ALT (**F**), AST (**G**), and TBIL (**H**) (n=3–5 mice per group). (**I**) Western blotting analysis of caspase-1 in the liver of HS mice pretreated with DMSO, VE, or Liproxstatin-1 and the corresponding statistical results (n =3) (**J**). (**K**) Measurement of plasma IL-1β content by ELISA (n=3 mice per group). Data are presented as the mean ± SEM. Significance was calculated using a one-way ANOVA with Tukey's *post hoc* test. Significance in (E) was calculated using the log-rank (Mantel-Cox) test.

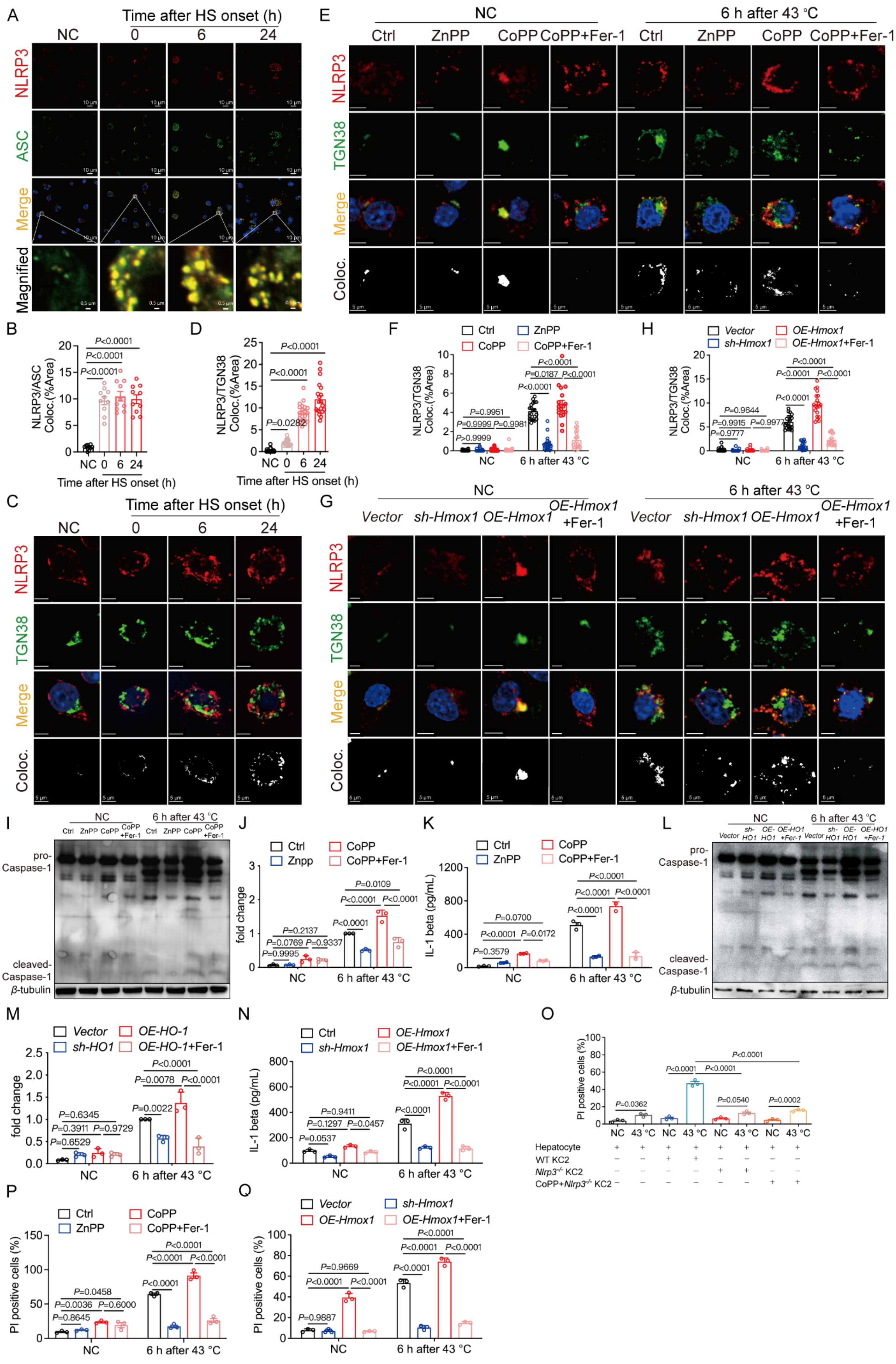
A
Time after HS onset (h)
NC
0
6
24
NLRP3
ASC
Merge
Magnified
B
NLRP3/ASC Coloc.(%Area)
P<0.0001
Time after HS onset (h)
D
NLRP3/TGN38 Coloc.(%Area)
P=0.0282
C
TGN38
Coloc.
E
6 h after 43 °C
Ctrl
ZnPP
CoPP
CoPP+Fer-1
F
P=0.9951
P=0.9999
P=0.9981
P>0.9999
P=0.0187
H
Vector
sh-Hmox1
OE-Hmox1
OE-Hmox1+Fer-1
P=0.9644
P=0.9915
P=0.9977
P=0.9777
G
I
pro-Caspase-1
cleaved-Caspase-1
β-tubulin
J
Znpp
fold change
P=0.0109
P=0.2137
P=0.0769
P=0.9337
P=0.9995
K
IL-1 beta (pg/mL)
P=0.0700
P=0.0172
P=0.3579
L
M
sh-HO1
OE-HO-1
OE-HO-1+Fer-1
P=0.6345
P=0.3911
P=0.9729
P=0.6529
P=0.0078
P=0.0022
N
P=0.9411
P=0.1297
P=0.0457
P=0.0537
O
PI positive cells (%)
P=0.0362
P=0.0540
P=0.0002
Hepatocyte
WT KC2
Nlrp3-/- KC2
CoPP+Nlrp3-/- KC2
P
P=0.0458
P=0.0036
P=0.6000
P=0.8645
Q
P=0.9669
P=0.9887

Figure S7. HMOX-1 activates NLRP3 inflammasome *in vitro*. (**A**) Representative immunofluorescence staining images for NLRP3 (red), ASC (green), and DAPI (blue) in KC2 treated at 43 °C for 3 h and then recovered at 37 °C for 0, 6, or 24 h (scale bar: 10 μm), and the corresponding statistical analysis (**B**) of co-localization of NLRP3 and ASC (*n*=10). (**C**) Representative immunofluorescence staining images for NLRP3 (red), TGN 38 (green), and DAPI (blue) in KC2 (scale bar: 5 μm), and the corresponding statistical analysis (*n*=20) (**D**). Representative immunofluorescence staining images (**E**) in KC2, pretreated with DMSO, ZnPP, CoPP, or CoPP+Fer-1 (scale bar: 5 μm), and the corresponding statistical analysis (*n*=20) (**F**). (**G**) Representative immunofluorescence staining images in *Vector*, *sh-Hmox1*, *OE-Hmox1*, or *OE-Hmox1*+Fer-1 of ImKCs (scale bar: 5 μm), and the corresponding statistical analysis (*n*=20) (**H**). (**I**) Western blotting analysis of caspase-1 in KC2 and the statistical analysis (*n*=3) (**J**). (**K**) Plasma IL-1*β* content measurement by ELISA (*n*=3). (**L**) Western blotting analysis of caspase-1 in ImKC and the statistical analysis (**M**) (*n*=3). (**N**) Plasma IL-1*β* content measurement by ELISA (*n*=3). (**O**) Cell death detection (PI positive) of hepatocytes (*n*=3). (**P**) Cell death detection (PI positive) of hepatocytes co-cultured with KC2 (*n*=3). (**Q**) Cell death detection (PI positive) of hepatocyte cell line AML12 co-cultured with ImKC cells (*n*=3). Summary data are presented as the mean ± SEM. Significance was calculated using a one-way ANOVA with Tukey's *post hoc* test.

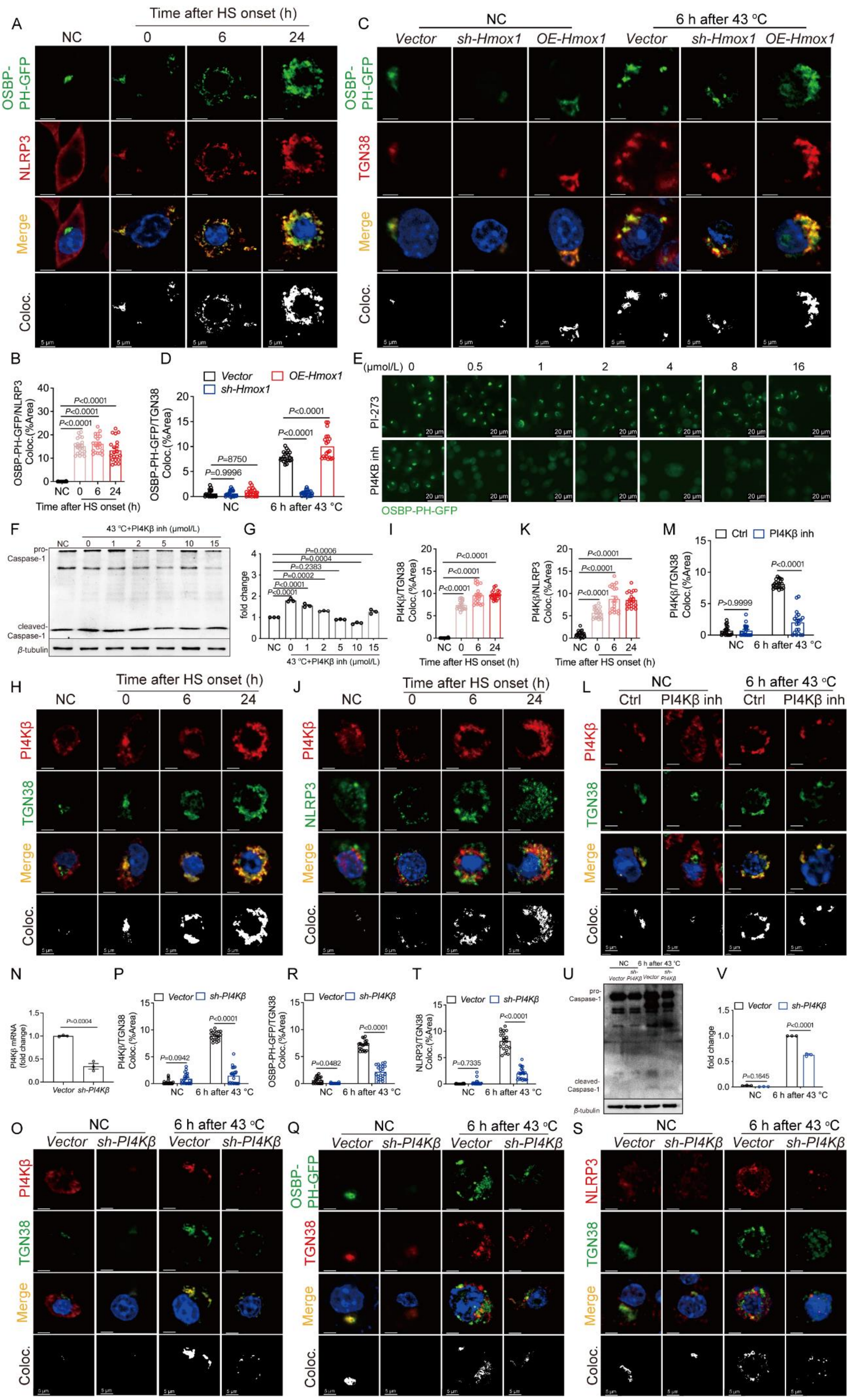

A
Time after HS onset (h)
NC
0
6
24
OSBP-PH-GFP
NLRP3
Merge
Coloc.
C
NC
6 h after 43 °C
Vector
sh-Hmox1
OE-Hmox1
TGN38
B
OSBP-PH-GFP/NLRP3 Coloc.(%Area)
Time after HS onset (h)
D
OSBP-PH-GFP/TGN38 Coloc.(%Area)
E
(μmol/L)
PI-273
PI4KB inh
OSBP-PH-GFP
F
43 °C+PI4Kβ inh (μmol/L)
pro-Caspase-1
cleaved-Caspase-1
β-tubulin
G
fold change
I
PI4Kβ/TGN38 Coloc.(%Area)
K
PI4Kβ/NLRP3 Coloc.(%Area)
M
Ctrl
PI4Kβ inh
H
PI4Kβ
TGN38
J
L
N
PI4Kβ mRNA (fold change)
sh-PI4Kβ
P
R
T
NLRP3/TGN38 Coloc.(%Area)
U
V
O
Q
S

Figure S8. PI4Kβ activates NLRP3 *in vitro*. (**A**) Representative immunofluorescence staining images for OSBP-PH-GFP (green), NLRP3 (red), and DAPI (blue) in KC2 treated at 43 °C for 3 h and recovered at 37 °C for 0, 6, or 24 h (scale bar: 5 μm), and the corresponding statistical analysis (**B**) of co-localization of OSBP-PH-GFP and NLRP3 (*n*=20). (**C**) Representative immunofluorescence staining images for OSBP-PH-GFP (green), TGN38 (red), and DAPI (blue) in *vector*, *sh-Hmox1*, or *OE-Hmox1*-treated ImKCs (scale bar: 5 μm), and the corresponding statistical analysis (*n*=20) (**D**). (**E**) Expression of PI4P (OSBP-PH-GFP, green) in KC2 pretreated with PI4K2A inhibitor PI-273 and PI4K*β* inhibitor (PI4K*β* inh) (scale bar: 20 μm). Western blotting analysis of caspase-1 in KC2 pretreated with DMSO or PI4K*β* inhibitor (**F**) and the statistical analysis (*n*=3) (**G**). (**H**) Representative immunofluorescence staining images for PI4K*β* (red), TGN38 (green), and DAPI (blue) in KC2 (scale bar: 5 μm), and the corresponding statistical analysis (**I**) (*n*=20). (**J**) Representative immunofluorescence staining images for PI4K*β* (red), NLRP3 (green), and DAPI (blue) in KC2 (scale bar: 5 μm), and the corresponding statistical analysis (**K**) (*n*=20). (**L**) Representative immunofluorescence staining images for PI4K*β* (red), TGN38 (green), and DAPI (blue) in KC2 (scale bar: 5 μm), pretreated with DMSO or PI4K*β* inhibitor, and the corresponding statistical analysis (*n*=20) (**M**). (**N**) Relative mRNA levels of *PI4Kβ* in *sh-PI4Kβ* ImKC stable transfected cell line with *PI4Kβ* knockdown (*n*=3). (**O**) Representative immunofluorescence staining images for PI4K*β* (red), TGN38 (green), and DAPI (blue) in *vector* or *sh-PI4Kβ*-treated cells (scale bar: 5 μm), and the corresponding statistical analysis (*n*=20) (**P**). (**Q**) Representative immunofluorescence staining images for OSBP-PH-GFP (green), TGN38 (red), and DAPI (blue) in *vector* or *sh-PI4Kβ*-treated cells (scale bar: 5 μm), and the corresponding statistical analysis (*n*=20) (**R**). (**S**) Representative immunofluorescence staining images for NLRP3 (red), TGN38 (green), and DAPI (blue) in *vector* or *sh-PI4Kβ*-treated cells (scale bar: 5 μm), and the corresponding statistical analysis (*n*=20) (**T**). (**U**) Western blotting analysis of caspase-1 in *vector* or *sh-PI4Kβ* of ImKCs and the statistical analysis (*n*=3) (**V**). Summary data are presented as the mean ± SEM. Significance was calculated using a one-way ANOVA with Tukey's *post hoc* test.

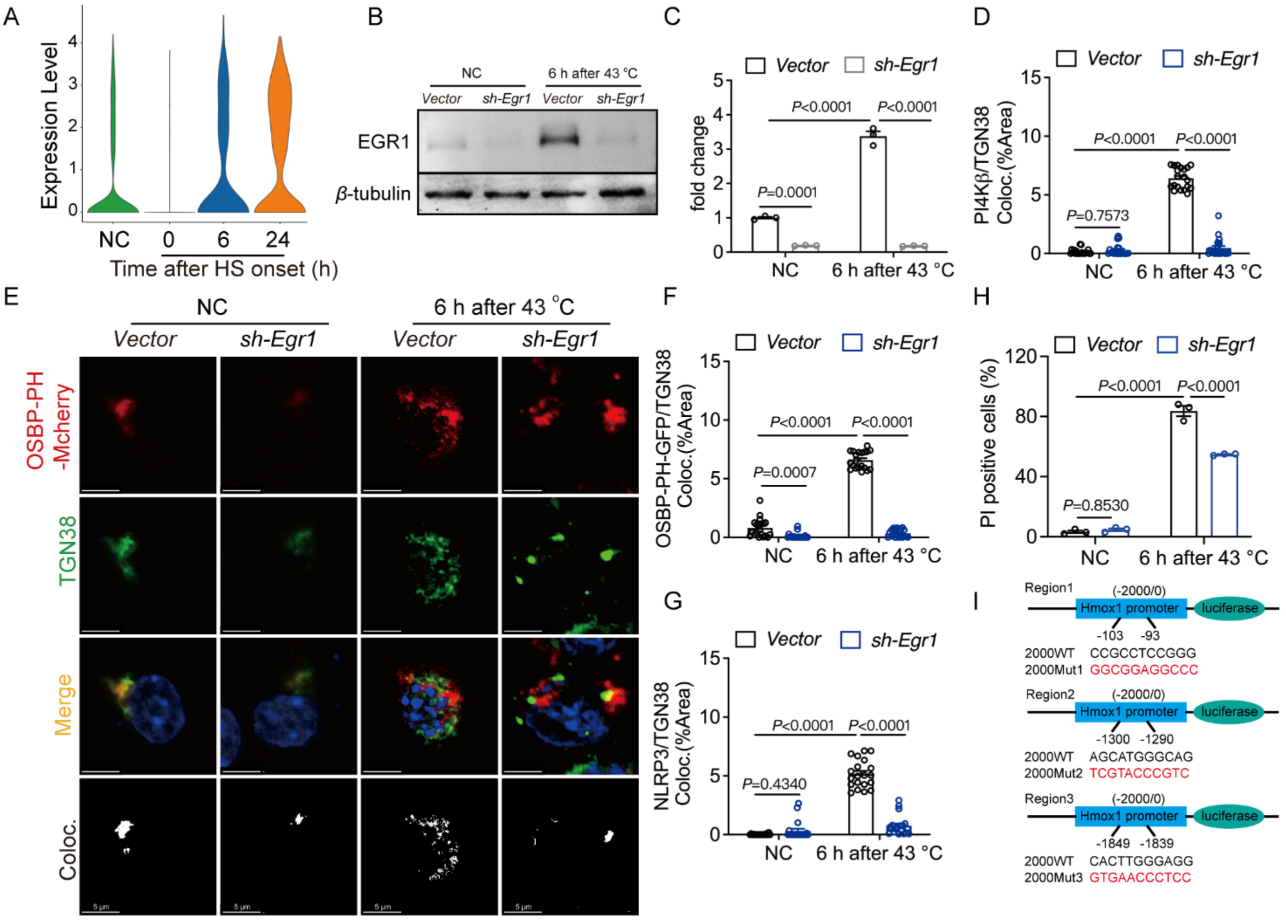

Figure S9. Up-regulation of EGR1 in KC2 following heat treatment. (**A**) Violin graph illustrating mRNA of *Egr1* in KC2. (**B**) Western blotting analysis of EGR1 in *vector* or *sh-Egr1*-treated cells and the corresponding statistical analysis (*n*=3) (**C**). (**D**) Statistical analysis (Fig. 7D) of co-localization of PI4K*β* and TGN38 (*n*=20). (**E**) Representative images of immunofluorescence staining for OSBP-PH-Mcherry (red), TGN38 (green), and DAPI (blue) (scale bar: 5 µm) and statistical analysis (*n*=20) (**F**). (**G**) Statistical analysis (Fig. 7E) of co-localization of NLRP3 and TGN38 (*n*=20). (**H**) Cell death detection (PI positive) (*n*=3). (**I**) Schematic diagram of site-directed mutagenesis of –103 – –93, –1300 – –1290, –1849 – –1839 sites in the *Hmox1* promoter region. Summary data are presented as the mean ± SEM. Significance was determined using a one-way ANOVA with Tukey's *post hoc* test.
